# *Streptococcus sanguinis* antagonizes *Prevotella melaninogenica* in the context of the cystic fibrosis respiratory microbiome

**DOI:** 10.1128/jb.00005-26

**Published:** 2026-02-27

**Authors:** Bassam El Hafi, Fabrice Jean-Pierre, Lily Taub, Thomas H. Hampton, George A. O'Toole

**Affiliations:** 1 Department of Microbiology and Immunology, Geisel School of Medicine at Dartmouth, Hanover, New Hampshire, USA; 2 Département de Biologie, Université de Sherbrooke 98630 , Sherbrooke, Québec, Canada; University of Notre Dame, Notre Dame, Indiana, USA

**Keywords:** *Prevotella*, *Streptococcus*, *Pseudomonas*, cystic fibrosis, microbiome, polymicrobial, nitric oxide

## Abstract

The latest generation of cystic fibrosis transmembrane conductance regulator-targeted modulator therapy, Elexacaftor/Tezacaftor/Ivacaftor (ETI), has significantly improved the clinical outcomes of persons with cystic fibrosis (pwCF) but does not appear to completely eliminate CF respiratory microbial colonization. Here, we analyzed over 4,200 publicly available respiratory microbiomes spanning pre- and post-ETI treatment periods and further stratified by age and type of sample. Our analyses reveal that despite reshaping of community composition by ETI, classical CF pathogens such as *Pseudomonas* and *Staphylococcus* spp. persist in sputum and sinonasal samples, while *Streptococcus* spp. drastically increase in prevalence and relative abundance post-ETI treatment. Obligate anaerobes such as *Prevotella* spp., *Fusobacterium* spp., *Porphyromonas* spp., and *Veillonella* spp. were readily detected post-ETI. We observed changes in the sputum samples from pwCF post-ETI, with an increase in the relative abundance of *Streptococcus* spp. and prevalence of *Prevotella* and a decline in *Pseudomonas* spp. Analysis of co-occurrence networks revealed mostly positive correlations between the different genera pre- and post-ETI; however, these correlations were reduced in number following ETI treatment, suggesting a disruption of community connectivity. To test one of the hypotheses raised by the computational analyses, we experimentally investigated the relationship between *Streptococcus sanguinis* and *Prevotella melaninogenica* in a CF sputum-like medium. Our data suggest that *S. sanguinis* uses reactive nitrogen species, and likely other factors, to antagonize *P. melaninogenica*, while *Pseudomonas aeruginosa* protects *P. melaninogenica* in CF-like culture conditions. Our findings implicate polymicrobial interactions, rather than individual species abundance, in determining microbial persistence and adaptation within the CF airway post-ETI.

**IMPORTANCE:**

The introduction of the latest cystic fibrosis transmembrane conductance regulator (CFTR)-targeted Elexacaftor/Tezacaftor/Ivacaftor (ETI) therapy represents a major therapeutic advance for persons with cystic fibrosis (pwCF); however, this therapy does not completely negate respiratory infections and colonization. We leverage large-scale publicly available microbiome data to demonstrate that while ETI therapy alters the respiratory microbial landscape, canonically prevalent and abundant CF pathogens persist in many pwCF and likely maintain ecological relevance through adaptive interactions with other taxa. Our *in vitro* findings also reveal that *Streptococcus sanguinis* can antagonize *Prevotella melaninogenica*, and that *Pseudomonas aeruginosa* can provide selective protection to quell this antagonism. These insights highlight the need to consider microbial interactions and community dynamics when evaluating long-term responses to CFTR modulators.

## INTRODUCTION

Chronic respiratory infections remain a major cause of morbidity and mortality in persons with cystic fibrosis (pwCF) (1). Historically, the bacterial species that have been most studied in the context of the CF lung have been *Pseudomonas aeruginosa* (*Pa*), *Staphylococcus aureus* (*Sa*), and members of the *Burkholderia cepacia* complex (2). Over the past decade, the increased utility of sequencing technologies and microbiome analyses in clinical and research settings has demonstrated that the lungs of pwCF are colonized by a variety of microorganisms that form polymicrobial communities composed of pathogenic as well as commensal species of varying prevalence and abundance (3–27).

The introduction of highly effective cystic fibrosis transmembrane conductance regulator (CFTR) modulator therapies, particularly the triple combination drug Trikafta (Elexacaftor/Tezacaftor/Ivacaftor [ETI]) in late 2019, has revolutionized care of pwCF by drastically reducing detrimental respiratory CF symptoms (28, 29). Within a year of ETI availability, the percentage of adults with CF treated for a pulmonary exacerbation at CF Care Centers in North America decreased from approximately 40% to about 18% (28, 30, 31), and has remained at around 15% annually (1, 32, 33). Meanwhile, the number of reported annual CF-related lung transplants, which had been gradually increasing since 2004, exhibited a steep decline from 249 transplants in 2019 to 61 transplants in 2024 (34). Despite the improvement in clinical outcomes, CF respiratory infections have not been completely eradicated following ETI treatment. Sputum samples from pwCF show an average decline in pathogen density post-ETI, but continue to be culture-positive over a 3 year period for organisms such as *P. aeruginosa*, *S. aureus*, *Burkholderia* spp., and *Stenotrophomonas maltophilia* for many pwCF (15, 27). Similarly, in the sinonasal cavity, microbiome studies have shown that *Pseudomonas* spp. abundance has decreased following ETI treatment but remains detectable by 16S rRNA amplicon sequencing as well as quantitative PCR (9, 24, 26). Therefore, understanding microbial dynamics in the context of CF respiratory symptoms post-ETI remains relevant.

Designing and validating experimental microbial model systems can aid us in understanding microbial interactions and physiology in relation to their environments as well as each other (35, 36). Previous work from our group developed a CF-relevant model polymicrobial community that is derived from the analysis of CF sputum samples and associated clinical metadata, and composed of the highly prevalent and abundant CF microbes *P. aeruginosa*, *S. aureus*, *Streptococcus sanguinis*, and *Prevotella melaninogenica* (*Pm*), cultured together in a medium that nutritionally resembles the pre-ETI CF lung environment (37–39). By testing microbial interactions within this community, we were able to identify an interesting relationship between *P. aeruginosa* and *P. melaninogenica* that involves cross-feeding TCA cycle intermediates by metabolizing mucin (40). However, we additionally observed a potentially antagonistic relationship between *P. melaninogenica* and *S. sanguinis* in this model (40), highlighting the complexity of microbial interactions in the airway of pwCF.

In this study, we analyzed multiple respiratory microbiome data sets derived from pwCF, which included samples prior to, as well as following the introduction of, ETI. This aggregated data set was stratified by ETI status first, then further stratified by age group or sample type. We observed that the respiratory microbiome of pwCF undergoes notable changes following ETI treatment based on sampling site along the respiratory tract, independent of age. We also noticed changes in the relative abundance of several genera, including *Streptococcus* spp. and *Prevotella* spp. post-ETI. Based on these observations, we tested a potential *Streptococcus-Prevotella in vitro* relationship using an in vitro model with CF-like conditions. We report that *S. sanguinis* is generally antagonistic toward *P. melaninogenica*, and that *P. aeruginosa* protects *P. melaninogenica* from this antagonism in CF-like culture conditions.

## RESULTS

### Collecting publicly available sequencing data sets to explore the changes in the respiratory microbiome of pwCF before and after modulator therapy

To gain a broader understanding of the microbial changes occurring in the context of CF respiratory infections and colonization following the introduction of the latest highly effective CFTR modulator therapy, we retrieved and analyzed publicly available sequencing data sets from multiple studies that spanned both pre- and post-ETI treatment periods (3–10, 12, 14, 15, 18, 19, 21, 23–25). In total, we analyzed 4,232 respiratory microbiome samples that were divided into pre-ETI (*n* = 3,897) and post-ETI (*n* = 335) data sets. The pre-ETI and post-ETI data sets were then subdivided by either age group or sample type (Table S1). The assigned age groups were either adult (≥18 years) or pediatric (<18 years). The sample types analyzed were sputum, bronchoalveolar lavage (BAL), and oropharynx for pre-ETI samples, and sputum, sinonasal, and oropharynx for post-ETI samples. The pre-ETI sample types also included an “Others” category (Table S1), which is comprised of nasal lavage, saliva, protected brush, and unlabeled sample types. This “Others” category was not included in the analysis because the sample types were either from a single study or were very few in sample number. Of note, the sample grouping according to age or sample type was manually assigned by mining the metadata associated with each study; therefore, not all samples were successfully assigned both an age group and sample type due to the limited information available for individual samples and variable levels of detail in reported study metadata.

### Classical CF pathogens persist at relatively high levels in the respiratory samples of adults with CF following ETI treatment

**Fig 1:**
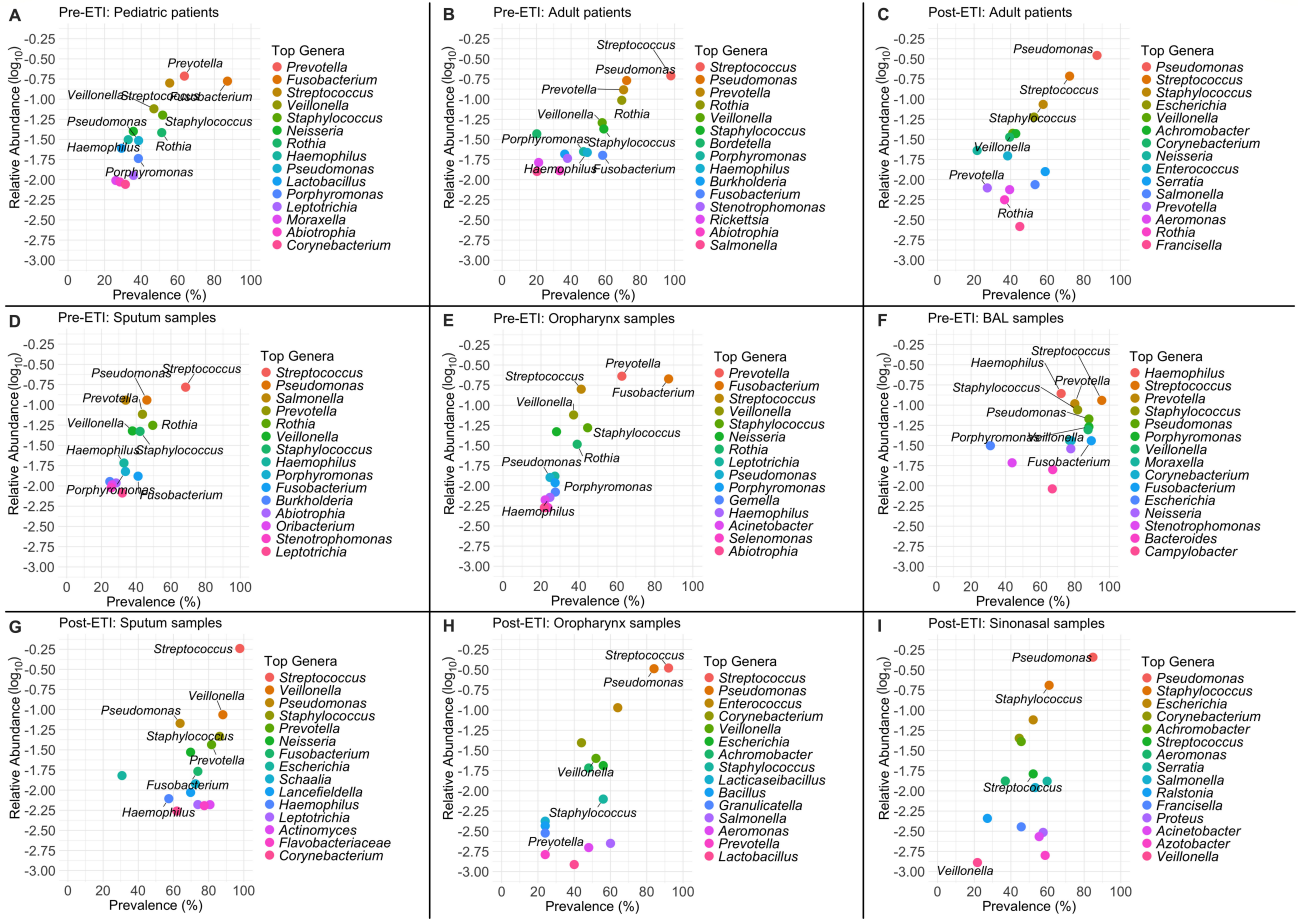
Determining the highest-ranking genera among pre- and post-ETI microbiome clinical samples using prevalence and relative abundance. (**A–I**) Scatter plots representing the relative abundance (log_10_) vs prevalence (%) of the top 15 most abundant and prevalent genera in our data set, which was stratified by pre- and post-ETI status first, then subdivided into age groups (pediatric <18 yo and adult >18 yo), inclusive of all sample types, or sample types (sputum, oropharynx, BAL, and sinonasal, as indicated), inclusive of all age groups.

By calculating the relative abundance and prevalence of the top 15 genera, which cover 86%–99% of cumulative abundance, in pre- and post-ETI data sets, stratified by age group and inclusive of all sample types, we observed that most of the organisms detected in the pre-ETI pediatric population were also detected in the pre-ETI adult population, particularly *Streptococcus*, *Prevotella*, *Pseudomonas*, *Fusobacterium*, *Rothia*, *Veillonella*, and *Staphylococcus*, with the major difference being the expected increased prevalence and relative abundance of *Pseudomonas* in pre-ETI adults versus the pre-ETI pediatric population (Fig. 1A and B). *Streptococcus* also showed an increase in both abundance and prevalence in pre-ETI adults versus the pediatric population (Fig. 1A and B).

Upon comparing the pre-ETI adult population to the post-ETI adult population, we observed that only *Streptococcus*, *Pseudomonas*, *Prevotella*, *Staphylococcus*, *Rothia*, *Veillonella*, and *Salmonella* were found in both groups among the 15 most prevalent microbes we analyzed (Fig. 1B and C). Meanwhile, organisms such as *Bordetella*, *Porphyromonas*, *Haemophilus*, *Burkholderia*, *Fusobacterium*, and *Stenotrophomonas* in the pre-ETI adult population were replaced by *Escherichia*, *Achromobacter*, *Corynebacterium*, *Neisseria*, *Enterococcus*, *Serratia*, and *Aeromonas* in the post-ETI adult population (Fig. 1B and C). Many of these microbes are not considered classic CF pathogens, and their relative abundance and prevalence are relatively low compared to organisms such as *Pseudomonas, Staphylococcus,* and the more abundant anaerobes. The most notable finding from this comparison was that *Pseudomonas* surpassed *Streptococcus* as the most prevalent and abundant community member in the post-ETI adult samples, while the obligate anaerobes, along with *Haemophilus* and *Stenotrophomonas*, generally exhibited reduced abundance and prevalence in these post-ETI adult samples (Fig. 1C). Of note, the post-ETI pediatric population in the available data sets was quite small (*n* = 8; Table S1) and originated from a single study. Thus, these data were not analyzed as a separate age group. Additional pediatric samples were included in another post-ETI study, but those samples could not be correctly assigned an age group due to the lack of sufficient metadata (15). Overall, these findings highlight the changing microbial ecology in the CF respiratory system (i.e., lung, sinonasal passage) as a function of ETI treatment status.

### *Pseudomonas* abundance is reduced in sputum samples following ETI treatment, but remains relatively high in oropharyngeal and sinonasal samples

Upon calculating the relative abundances and prevalence of the top 15 genera in pre- and post-ETI data sets, stratified by sample type and encompassing all age groups, we observed that the microbial communities from the various sampling sites exhibited different responses to ETI therapy (Fig. 1D through I). When comparing sputum samples pre- and post-ETI, we observed an increase in the prevalence of several genera, including *Streptococcus*, *Staphylococcus*, *Veillonella*, *Pseudomonas*, *Prevotella*, and *Fusobacterium* post-ETI. *Streptococcus* additionally exhibited the sharpest increase in relative abundance (Fig. 1D and G). The highest-ranking genera in post-ETI sputum were *Streptococcus*, *Veillonella*, *Pseudomonas*, *Staphylococcus*, and *Prevotella* (Fig. 1G).

When comparing the pre- and post-ETI oropharyngeal samples, we observed that 10 of the top 15 genera pre-ETI were replaced by other organisms post-ETI. Most notably, the anaerobic oral commensal genera *Fusobacterium*, *Rothia*, and *Porphyromonas* (41–43) were no longer among the top oral genera post-ETI. Instead, *Enterococcus*, *Corynebacterium*, *Escherichia*, and *Achromobacter* emerged (Fig. 1E and H). On the other hand, *Streptococcus*, *Pseudomonas*, *Staphylococcus*, *Veillonella*, and *Prevotella* were the only genera found in both data sets, with *Streptococcus* and *Pseudomonas* exhibiting an increase in their prevalence and relative abundance levels in these oropharyngeal samples, while *Staphylococcus* and *Prevotella* declined in both metrics (Fig. 1E and H).

For some of the analyses, there were no samples from the same respiratory regions pre- and post-ETI, thus we could make only a limited set of comparisons. For example, when analyzing the pre-ETI BAL samples, we observed 60% overlap in the top genera with those identified in the pre-ETI sputum samples, with *Haemophilus*, *Streptococcus*, *Prevotella*, *Staphylococcus*, and *Pseudomonas* having highest prevalence and relative abundances (Fig. 1F). For the post-ETI sinonasal samples, we observed 60% overlap in the top genera with those of the post-ETI oropharyngeal samples (Fig. 1H and I). However, the sinonasal samples exhibited varying relative abundance and prevalence levels, with *Pseudomonas* being the highest in both metrics, followed by *Staphylococcus*, *Escherichia*, *Corynebacterium*, and *Achromobacter* (Fig. 1I).

### Microbial co-occurrence networks reveal predominantly positive interactions that are altered with ETI treatment in the different sample sites

**Fig 2:**
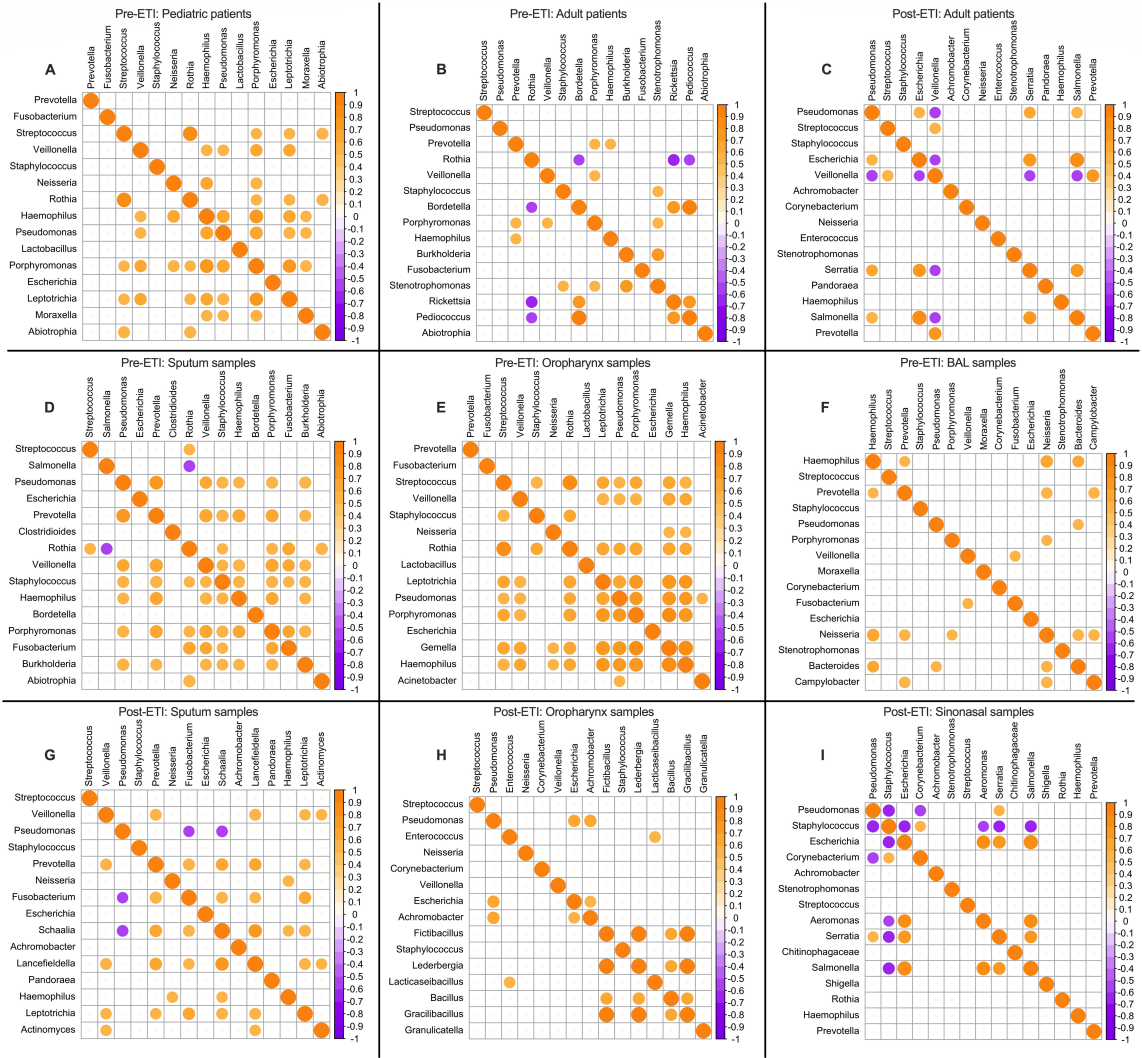
Correlations among top genera of pre- and post-ETI clinical samples. (**A–I**) Heatmaps representing the calculated Spearman correlation matrices of the top 15 most abundant genera in each of the data sets. Only strong (|r_s_| > 0.5) and significant (*P*-value <0.05) correlations that may be biologically relevant were plotted. The scale shows the Spearman correlation coefficient from 1 (orange) to −1 (purple).

In addition to determining the top 15 genera in our CF respiratory samples data sets based on their prevalence and relative abundance levels, we generated Spearman correlation matrices of the top 15 genera by relative abundance levels only (Table S2) for network analysis to identify whether the genera with the highest relative abundance exhibit positive or negative correlations with one another. This analysis provides a prediction of potential microbial interactions within communities (44, 45). Our findings were visualized by heatmaps and co-occurrence networks where only the strong (|r_s_| > 0.5) and statistically significant (*P*-value <0.05) Spearman correlations were plotted. When looking at the data, we did not observe consistent correlation trends across age groups, sample types, and ETI status. For those correlations that were identified, most were positive, with only a few scattered negative correlations, for example, *Rothia*, *Veillonella*, *Pseudomonas*, or *Staphylococcus* (Fig. 2A through I, purple dots). It was interesting to note that even though some genera such as *Pseudomonas*, *Staphylococcus*, *Streptococcus*, *Prevotella*, *Veillonella*, and *Haemophilus* were included in almost all data sets, they still exhibited different correlation strengths and numbers of connections among each other across the different sample types. Another observation was that ETI appears to have generally lowered the number of significant positive microbial correlations of the top genera (without increasing the number of negative correlations), irrespective of their identities, when comparing both sputum and oropharyngeal samples pre- and post-ETI (Fig. 2D, E, G, and H).

**Fig 3:**
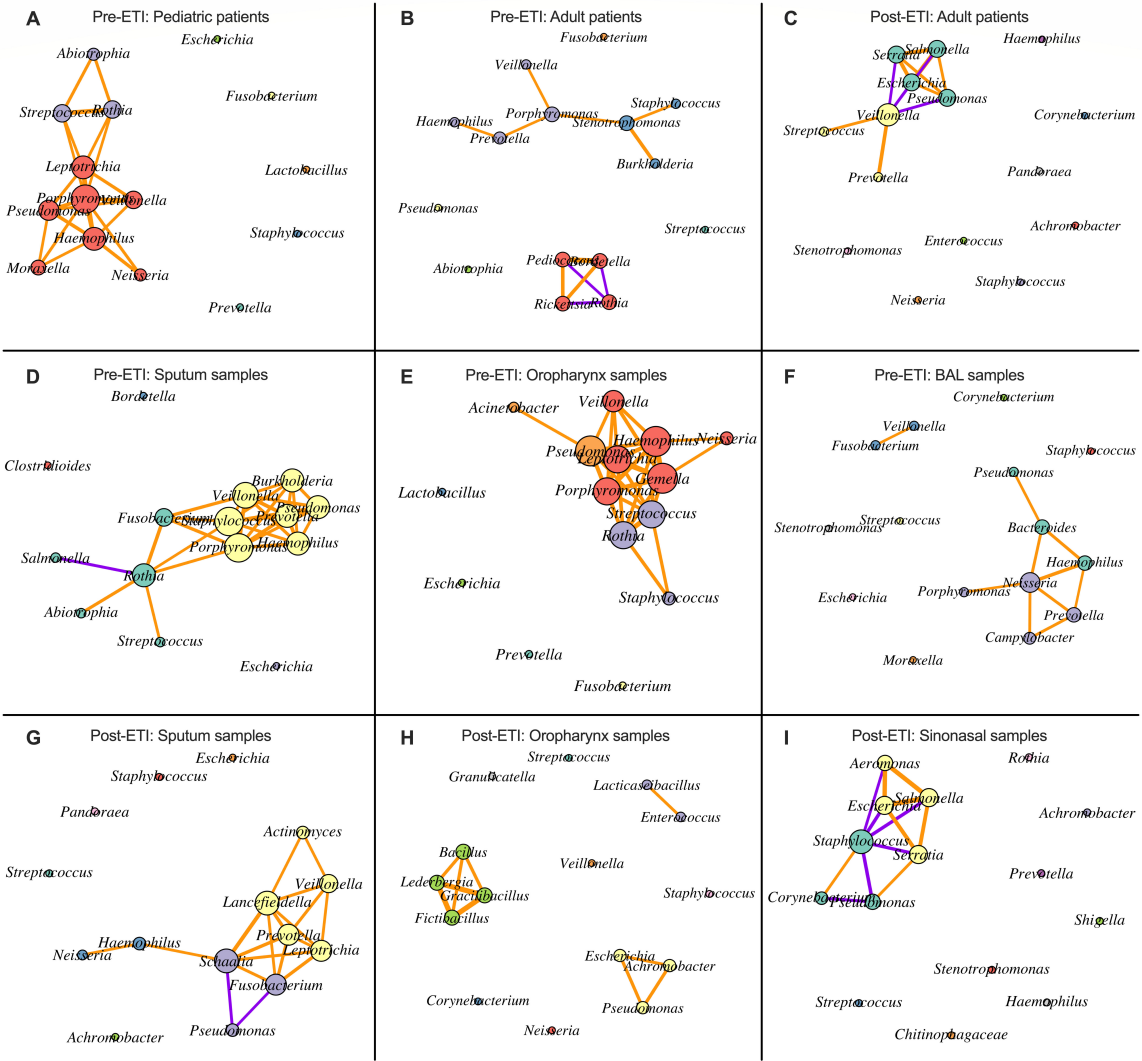
Co-occurrence networks among top genera of pre- and post-ETI clinical samples. (**A–I**) Co-occurrence networks derived from the Spearman correlation matrices in Table S2. Nodes in the network represent genera, while the connections with other nodes signify correlations. Only strong (|r_s_| > 0.5) and significant (*P*-value <0.05) correlations that may be biologically relevant were plotted. The color of the connectors represents either positive (orange) or negative (purple) correlations. The color of each node represents the community with which that genus co-occurs.

Upon analyzing the co-occurrence networks of the pre-ETI pediatric and adult patient samples, we noticed that *Porphyromonas* was predicted to be a hub node in both data sets by having the most connections to other genera (Fig. 3A and B). *Rothia*, on the other hand, was in a positive correlation cluster with *Streptococcus* and *Abiotrophia* in the pre-ETI pediatric samples, but is part of a cluster with negative correlations to *Rickettsia*, *Pediococcus*, and *Bordetella*, which were all positively correlated to one another, in the pre-ETI adult samples even though *Streptococcus* and *Abiotrophia* were still highly abundant in these latter samples (Fig. 3A and B). *Pseudomonas* and *Streptococcus* were part of two separate clusters in the pre-ETI pediatric patient samples; however, they were isolated genera in the pre-ETI adult patient samples even though their previous correlation partners were still among the most abundant genera (Fig. 3A and B). *Staphylococcus* and *Prevotella* exhibited the opposite phenomenon, where they are part of the same cluster in the pre-ETI adult samples but isolated genera in the pre-ETI pediatric samples (Fig. 3A and B).

By comparing the pre-ETI adult samples to the post-ETI adult samples, we observed that *Veillonella*, which was only correlated positively with *Porphyromonas* in the pre-ETI adult samples, was predicted to be a hub node in the post-ETI adult samples, and was positively correlated with *Streptococcus* and *Prevotella*, and negatively correlated with *Pseudomonas*, *Serratia*, *Salmonella*, and *Escherichia*, which were in turn all positively correlated with each other (Fig. 3B and C). *Porphyromonas* was no longer among the top genera in the post-ETI adult samples.

By comparing the microbial network results from the sputum samples across pre- and post-ETI data sets, we observed a positive correlation cluster, with *Porphyromonas* and *Staphylococcus* as predicted hub nodes, and which included *Prevotella*, *Pseudomonas*, *Haemophilus*, *Veillonella*, and *Burkholderia* in the pre-ETI sputum data set (Fig. 3D). The major correlation cluster in the post-ETI sputum samples was smaller and composed of *Prevotella* and *Schaalia* as the predicted hub nodes, and these organisms were positively correlated with *Veillonella*, *Lancefieldella*, *Leptotrichia*, and *Fusobacterium* (Fig. 3G). *Staphylococcus* and *Streptococcus* were no longer part of a cluster in the post-ETI sputum samples, and *Pseudomonas* showed a negative correlation with *Fusobacterium* and *Schaalia* in these post-ETI sputum samples (Fig. 3G).

Comparing microbial network results from the oropharyngeal samples across pre- and post-ETI data sets, we observed tight clustering of multiple genera in the pre-ETI samples with *Pseudomonas*, *Porphyromonas*, and *Streptococcus* predicted to be hub nodes to three positive correlation clusters (Fig. 3E). On the other hand, the post-ETI oropharyngeal samples revealed a more disconnected network with only two small clusters (Fig. 3H).

When analyzing the microbial network of the pre-ETI bronchoalveolar lavage samples, we observed mostly isolated genera with two small clusters. *Neisseria* was the predicted hub node connecting the two clusters. The first cluster was composed of *Prevotella*, *Campylobacter*, and *Porphyromonas*, while the second cluster was composed of *Haemophilus*, *Bacteroides*, and *Pseudomonas* (Fig. 3F). *Veillonella* and *Fusobacterium* were also positively correlated. When analyzing the microbial network of the post-ETI sinonasal samples, we observed mostly isolated genera; however, *Staphylococcus* was predicted to be a hub node that was negatively correlated with several other members, including *Pseudomonas*, *Serratia*, *Salmonella*, *Escherichia*, and *Aeromonas* (Fig. 3I).

These observations demonstrate that the CF respiratory system is heterogeneous and susceptible to changing relationships correlated with ETI treatment, in particular with a reduction in network connections post-treatment. Further, network analysis predicts that the most abundant bacteria are the ones that seem capable of adapting to these changes by altering interaction networks, with positively correlated interactions prevailing in general despite the reduction in the overall number of connections. In the following sections, we use our model microbial community to interrogate some of these predicted interactions and the mechanisms that may drive them.

### *S. sanguinis* antagonizes *P. melaninogenica* in CF and non-CF sputum-like settings, but *P. aeruginosa* can provide limited protection

One observation from the analyses above is that in sputum samples, the prevalence and relative abundance of *Streptococcus* is increased in post-ETI samples (Fig. 1D and G). For *Prevotella* spp. post-ETI, while the prevalence of this organism increases by 36%, its relative abundance is reduced by approximately half (Fig. 1D and G). Furthermore, pre-ETI there is a positive correlation between *Streptococcus* and *Prevotella*, which is lost post-ETI. Furthermore, we previously reported that *P. melaninogenica* cannot be recovered when grown anoxically in mucin-containing artificial sputum medium (ASM) as a monoculture, but viable *Prevotella* can be detected when grown as part of a four-species polymicrobial community (including *P. aeruginosa* PA14, *S. aureus* Newman, and *S. sanguinis* SK36) that was designed based on the communities found in the CF sputum microbiome and associated metadata (37, 38). Taking together our computational and experimental data, it appears that the relationship among these organisms is complex and reflects interactions among more than two microbes. We test these ideas below.

**Fig 4:**
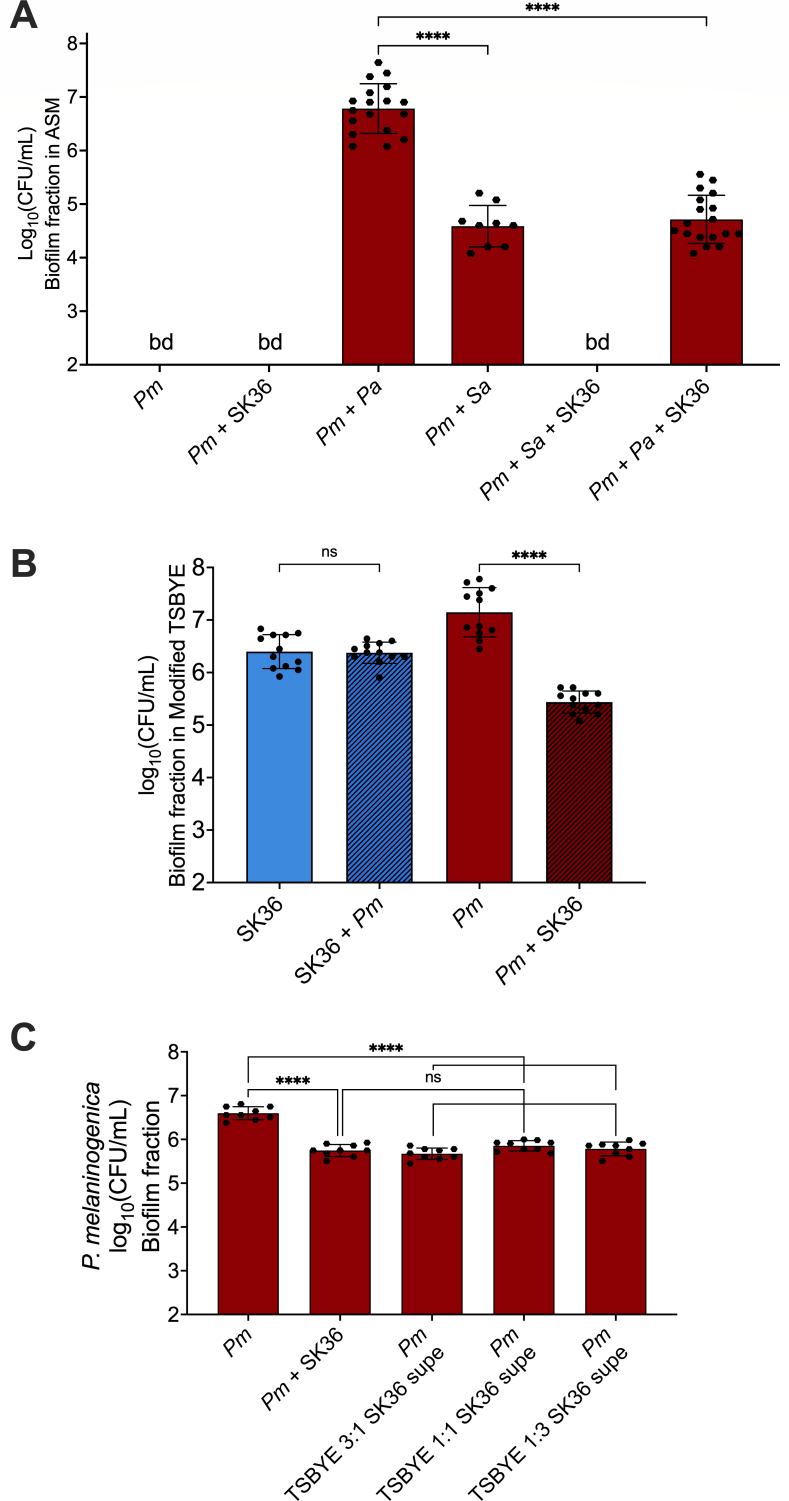
Biofilm co-culture assays. (**A**) All cultures were performed using mucin-containing ASM under anoxic growth conditions at 37°C for 24 h. The viable counts of the biofilm fraction of the co-cultures are plotted, showing the viable counts of *Pm* in different combinations with *P. aeruginosa* PA14 (*Pa*), *Sa*, and *S. sanguinis* SK36 (SK36). Statistical significance was calculated using ordinary one-way analysis of variance (ANOVA) with Tukey’s multiple comparisons test. ****, *P* < 0.0001. Additional statistical comparisons can be found in Table S4. (**B**) All cultures were performed using modified TSBYE under anoxic growth conditions at 37°C for 24 h. The viable counts of the biofilm fraction of the co-cultures are plotted, showing the mono- and co-culture viable counts of *S. sanguinis* SK36 (SK36) in blue and *Pm* in red. Statistical significance was calculated using ordinary one-way ANOVA with Tukey’s multiple comparisons test. ns = not significant and ****, *P* < 0.0001. Additional statistical comparisons can be found in Table S4. (**C**) All cultures were performed using modified TSBYE under anoxic growth conditions at 37°C for 24 h. The viable counts of the biofilm fraction plotted were of *P. melaninogenica* as a monoculture, in co-culture with *S. sanguinis*, or as a monoculture supplemented with increasing concentrations of *S. sanguinis* cell-free supernatants. Statistical significance was calculated using ordinary one-way ANOVA with Tukey’s multiple comparisons test. ns = not significant and ****, *P* < 0.0001. Additional statistical comparisons can be found in Table S4.

We have shown that *P. aeruginosa* plays a critical role in supporting the growth of *P. melaninogenica* in our four-microbe model community (40). In fact, co-culturing *Pm* and *Pa* alone in ASM under anoxic conditions was sufficient to recapitulate the growth phenotype of *P. melaninogenica* that was observed with the four-species community, as previously reported (40) and demonstrated here to reach ~10^7^ colony forming units (CFU)/mL (Fig. 4A, 3rd bar). Co-culturing *P. melaninogenica* and *Sa* in the same conditions also allowed for the detection of *P. melaninogenica* in the ASM following incubation for 24 h, although only to 4 × 10^4^ CFU/mL (Fig. 4A, 4th bar), which was significantly lower than the *P. melaninogenica-P. aeruginosa* co-culture at 6.3 × 10^6^ CFU/mL. On the other hand, co-culturing *P. melaninogenica* with *S. sanguinis* SK36 in the same culture conditions did not support the growth of *P. melaninogenica* (Fig. 4A, 2nd bar) and adding *S. aureus* as a third species in the culture did not contribute to the survival of *P. melaninogenica* in this system (Fig. 4A, 5th bar). When *P. aeruginosa* was added to the mixture with *P. melaninogenica* and *S. sanguinis*, *P. melaninogenica* was detectable at 5 × 10^4^ CFU/mL after incubation, which was still significantly lower than the *P. melaninogenica-P. aeruginosa* dual culture (Fig. 4A, 6th bar). These observations lead us to conclude that *S. sanguinis* might be inhibiting and/or killing *P. melaninogenica* in co-culture under CF-like conditions, and that *P. aeruginosa*, but not *S. aureus*, provides a form of limited protection to *P. melaninogenica* from *S. sanguinis* in mucin-containing ASM during anoxic growth. It is important to note that the viability of *S. sanguinis*, *P. aeruginosa*, and *S. aureus* was largely unaffected by the different culture conditions and combinations compared to their monocultures, except for *S. sanguinis* exhibiting a modest, but significantly higher growth in mixed culture with *P. aeruginosa* and *P. melaninogenica* compared to its monoculture (Fig. S1A). This latter finding was not an unexpected outcome given that *S. sanguinis* has been previously reported to exhibit better growth when co-cultured with *P. aeruginosa* compared to monoculture (46, 47).

To test the hypothesis that *S. sanguinis*-mediated antagonism of *P. melaninogenica* was not restricted to ASM, we co-cultured both organisms using the same method and culture conditions as above; however, we replaced ASM with modified TSBYE, a medium that supports the growth of *P. melaninogenica* as a monoculture. Following a 24 h incubation, it was evident that *P. melaninogenica* was significantly less recoverable (by ~10-fold) in co-culture with *S. sanguinis* compared to monoculture (Fig. 4B, right, red bars). *S. sanguinis* was unaffected by the presence of *P. melaninogenica* (Fig. 4B, left, blue bars). To test whether *S. sanguinis* was antagonizing *P. melaninogenica* through secreted products, we grew *P. melaninogenica* as a monoculture in modified TSBYE supplemented with increasing concentrations of spent, cell-free *S. sanguinis* supernatant from modified TSBYE-grown cells collected after 6 h of incubation. The 6 h time point was selected based on data presented below. We observed that the recovery of *P. melaninogenica* in the *P. melaninogenica-S. sanguinis* co-culture compared to *P. melaninogenica* monocultures with added *S. sanguinis* supernatant was not significantly different (Fig. 4C). Both these tested conditions resulted in significantly lower viability than the *P. melaninogenica* monoculture control with modified TSBYE (Fig. 4C). Therefore, it appears that *S. sanguinis* antagonizes *P. melaninogenica* through secreted products that can be produced under non-CF-like medium conditions.

### *S. sanguinis* antagonism versus *P. melaninogenica* is not mediated by H_2_O_2_ production, and *P. aeruginosa* does not protect *P. melaninogenica* against *S. sanguinis* in all culture conditions

**Fig 5:**
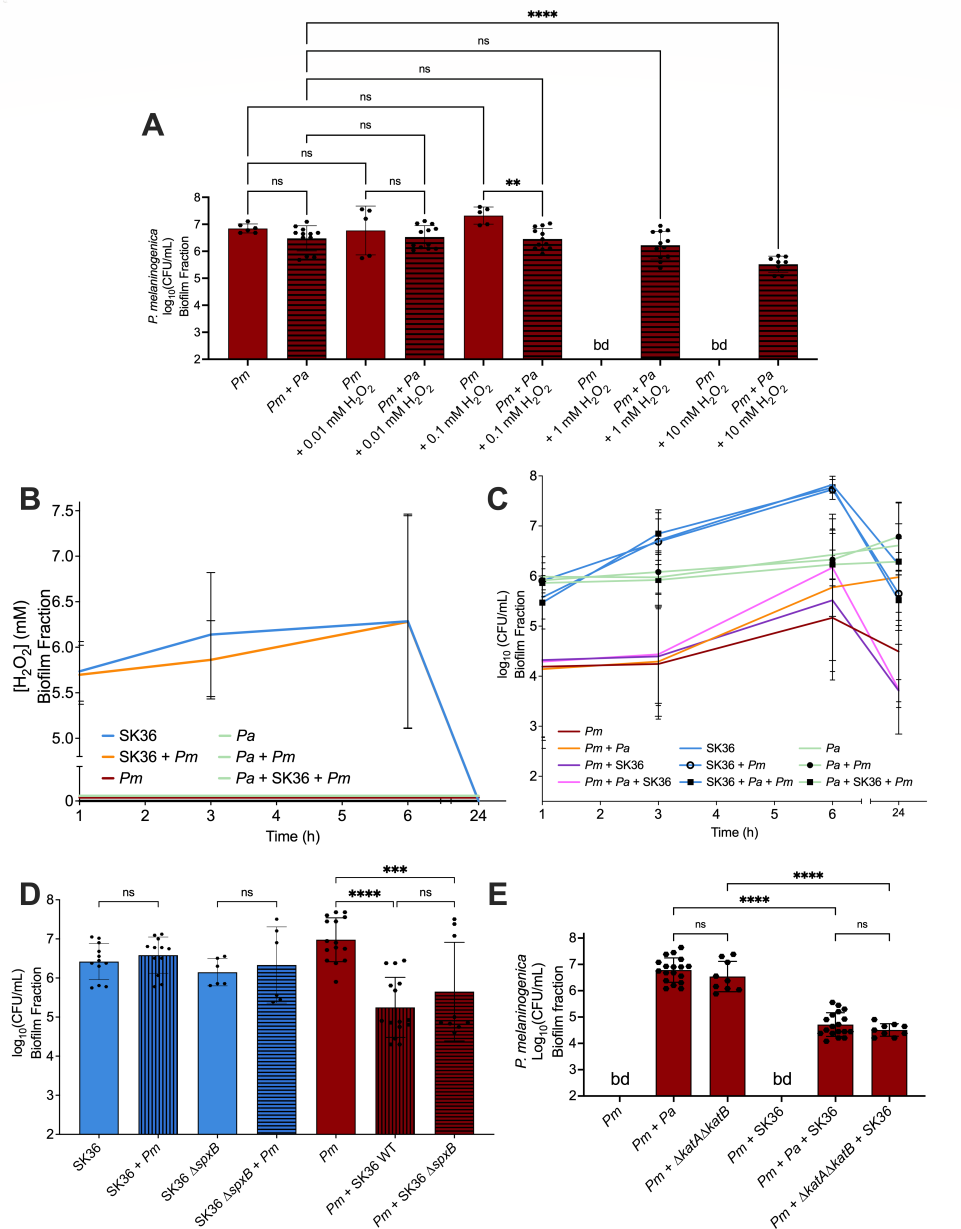
Impact of oxidative stress on biofilm microbial interactions. (**A**) The viable counts of the biofilm fraction of *Pm* when cultured in modified TSBYE under anoxic growth conditions at 37°C for 24 h and exposed to increasing concentrations of H_2_O_2_ as either a monoculture or in co-culture with *Pa*. Statistical significance was calculated using ordinary one-way ANOVA with Tukey’s multiple comparisons test. ns = not significant; **, *P* < 0.005; and ****, *P* < 0.0001. Additional statistical comparisons can be found in Table S4. (**B**) The measured concentration of hydrogen peroxide in the biofilm fraction of several culture conditions that include different combinations of *Pm*, *Pa*, and *S. sanguinis* (SK36) in modified TSBYE at 37°C over a 24 h time course with sampling times of 1, 3, 6, and 24 h. (**C**) The viable counts of the biofilm fraction from culture conditions that include different combinations of *Pm*, *Pa*, and *S. sanguinis* (SK36) in modified TSBYE at 37°C over a 24 h time course with sampling times of 1, 3, 6, and 24 h. (**D**) The viable counts of the biofilm fraction of *Pm*, *S. sanguinis* SK36 (S36), and/or *S. sanguinis* SK36 Δ*spxB* (SK36 Δ*spxB*) in monoculture and co-culture as indicated in modified TSBYE under anoxic growth conditions at 37°C for 24 h. Statistical significance was calculated using ordinary one-way ANOVA with Tukey’s multiple comparisons test. ***P = 0.0001, ****P < 0.0001, ns = not significant. Additional statistical comparisons can be found in Table S4. (**E**) The viable counts in the biofilm fraction from culture conditions that include different combinations of *Pm*, *P. aeruginosa* PA14 (*Pa*), *P. aeruginosa* PA14 Δ*katA*Δ*katB* (Δ*katA*Δ*katB*), and *S. sanguinis* (SK36) in modified mucin-containing ASM at 37°C for 24 h. Statistical significance was calculated using ordinary one-way ANOVA with Tukey’s multiple comparisons test. ns = not significant and ****, *P* < 0.0001. Additional statistical comparisons can be found in Table S4.

To further characterize the relationship between *P. melaninogenica* and *S. sanguinis*, we hypothesized that *S. sanguinis* was antagonizing *P. melaninogenica* through oxidative stress via hydrogen peroxide production. This prediction was based on the prior observation that *S. sanguinis* antagonizes other organisms, including other species of *Streptococcus*, by inducing oxidative stress (48–51). Therefore, to test whether *P. melaninogenica* can withstand the potential oxidative stress inflicted by *S. sanguinis*, and whether *P. aeruginosa* can rescue the viability of *P. melaninogenica*, we grew *P. aeruginosa* and *P. melaninogenica* anoxically as monocultures and in co-culture for 24 h in modified TSBYE with increasing concentrations of H_2_O_2_ (0.01 → 10 mM). We observed that *P. melaninogenica* was not detected in monoculture containing ≥1 mM H_2_O_2_ (Fig. 5A). However, *P. melaninogenica* was detected in cultures containing up to 10 mM H_2_O_2_ when co-cultured with *P. aeruginosa* (Fig. 5A). Therefore, *P. melaninogenica* is susceptible to millimolar levels of peroxide, and *P. aeruginosa* can help *P. melaninogenica* survive such stress. Of note, *P. aeruginosa* was able to survive H_2_O_2_ stress in this assay up to 10 mM H_2_O_2_ without significant changes in viability under any of the tested culture conditions (Fig. S1B).

To determine the concentration of H_2_O_2_ that *P. melaninogenica* is exposed to when co-cultured with *S. sanguinis*, we measured the concentration of H_2_O_2_ produced during the anoxic co-culture of *P. melaninogenica* with *S. sanguinis* over a 24 h time course. Due to technical reasons associated with determining hydrogen peroxide concentrations using the commercial kit to measure this molecule, as stated in the Materials and Methods section, the medium used for these co-culture time course assays was modified TSBYE, rather than mucin-containing ASM. However, as we show above, *S. sanguinis* appeared antagonistic toward *P. melaninogenica* in modified TSBYE (Fig. 4B).

Mono- and co-cultures of *P. melaninogenica* and *S. sanguinis* were incubated in parallel with mono- and co-cultures of *P. melaninogenica* and *P. aeruginosa* as control conditions. Additionally, a medium blank control was also included in the assay to ensure that any recorded hydrogen peroxide was not due to medium components. We report that H_2_O_2_ was measurable in the biofilm fraction at a concentration range of 5.75 mM–6.25 mM during the first 6 h of culture in the *S. sanguinis* monoculture condition as well as the *S. sanguinis-P. melaninogenica* co-culture condition (Fig. 5B). However, H_2_O_2_ was not detectable in the *P. melaninogenica* monoculture as well as any of the conditions that contained *P. aeruginosa*, including the triple culture of *P. aeruginosa*, *P. melaninogenica,* and *S. sanguinis* (Fig. 5B). Therefore, it can be concluded that *P. aeruginosa* reduced H_2_O_2_ levels in the biofilm fraction either by eliminating the hydrogen peroxide that was produced by *S. sanguinis* or inhibiting *S. sanguinis* from producing hydrogen peroxide in co-cultures. These observations also suggest that *S. sanguinis-*produced H_2_O_2_ could result in reduced *P. melaninogenica* viability in co-culture. However, by enumerating the CFU from the biofilm fractions of these time course co-cultures, we noticed that the final biofilm CFU/mL of *P. melaninogenica* when co-cultured with *S. sanguinis* was almost identical to that of the *P. melaninogenica* triple culture with *S. sanguinis* and *P. aeruginosa*, at around 5 × 10^3^ CFU/mL (Fig. 5C), even though the triple culture did not have detectable H_2_O_2_ at any point during the time course (Fig. 5B). Additionally, if we plot the log_10_ (CFU/mL) values of *P. melaninogenica* in co-culture with *S. sanguinis* against the concentration of H_2_O_2_ in the same co-culture, we observe a nearly flat linear regression trend line (Fig. S1C), indicating no correlation between the two factors. To further interrogate the involvement of hydrogen peroxide in the interaction between *P. melaninogenica* and *S. sanguinis*, we co-cultured *P. melaninogenica* with a *S. sanguinis* Δ*spxB* mutant (52) in modified TSBYE. *S. sanguinis* produces hydrogen peroxide as a byproduct of the conversion of pyruvate into acetyl phosphate, which is a reaction catalyzed by pyruvate oxidase, encoded by *spxB* (53). We observe that the viability of *P. melaninogenica* in co-culture with the *S. sanguinis* Δ*spxB* is not significantly different than its viability when co-cultured with wild-type *S. sanguinis*, and both are significantly reduced compared to *P. melaninogenica* grown as a monoculture in modified TSBYE (Fig. 5D).

It is important to note that neither *S. sanguinis* nor *P. aeruginosa* was affected by the different culture conditions and co-cultures through the time course. We observed that *S. sanguinis*, irrespective of its culture condition, grew to around 5 × 10^7^ CFU/mL by the 6th hour before declining to reach approximately 10^6^ CFU/mL by the 24th hour (Fig. 5C). *P. aeruginosa*, on the other hand, exhibited consistent growth throughout the time course to reach a final viable count of ~ 3 × 10^6^ CFU/mL across all conditions by the endpoint (Fig. 5C). Based on these observations, we conclude that *S. sanguinis* might be inhibiting the growth of *P. melaninogenica* in co-culture through mechanisms that do not only rely on oxidative stress via hydrogen peroxide.

To understand whether *P. aeruginosa* was removing hydrogen peroxide from the co-culture medium or hindering its production by *S. sanguinis*, we co-cultured *P. melaninogenica* and *S. sanguinis* in ASM under anoxic growth conditions with a *P. aeruginosa* ∆*katA*∆*katB* double deletion mutant that should be incapable of producing catalase, and thus, would not be able to optimally remove hydrogen peroxide from its environment (54). We first noted that *P. aeruginosa* Δ*katA*Δ*katB* supported the growth of *P. melaninogenica* in ASM to the same extent as wild-type *P. aeruginosa*, which was around 3.2 × 10^6^ CFU/mL (Fig. 5E). *P. melaninogenica* was again undetectable in co-culture with *S. sanguinis* (Fig. 5E) but was recoverable to about 3 × 10^4^ CFU/mL when grown with *S. sanguinis* and wild-type *P. aeruginosa* (Fig. 5E). If *P. aeruginosa* was protecting *P. melaninogenica* from *S. sanguinis* by eliminating H_2_O_2_, then co-culturing both *P. melaninogenica* and *S. sanguinis* with the catalase-deficient *P. aeruginosa* Δ*katA*Δ*katB* mutant would likely not result in detectable viable *P. melaninogenica*. However, we observed that *P. melaninogenica* was recoverable to approximately 3 × 10^4^ CFU/mL after co-culture with *S. sanguinis* and *P. aeruginosa* Δ*katA*Δ*katB* (Fig. 5E), which was nearly identical to the *P. melaninogenica* CFU/mL recorded in the triple culture with *S. sanguinis* and wild-type *P. aeruginosa* (Fig. 5E).

Taking these data together, while the levels of H_2_O_2_ are lower in the presence of *P. aeruginosa*, indicating potential protection of *P. melaninogenica* from oxidative stress through hydrogen peroxide elimination, we conclude that the observed antagonism of *S. sanguinis* against *P. melaninogenica* in co-culture is potentially the result of mechanisms other than oxidative stress via hydrogen peroxide given the lack of correlation between H_2_O_2_ levels and *P. melaninogenica* viability, as well as the lack of impact of the mutant strains tested here.

### Reactive nitrogen species produced by *S. sanguinis* may be contributing to its antagonism against *P. melaninogenica* in co-culture

**Fig 6:**
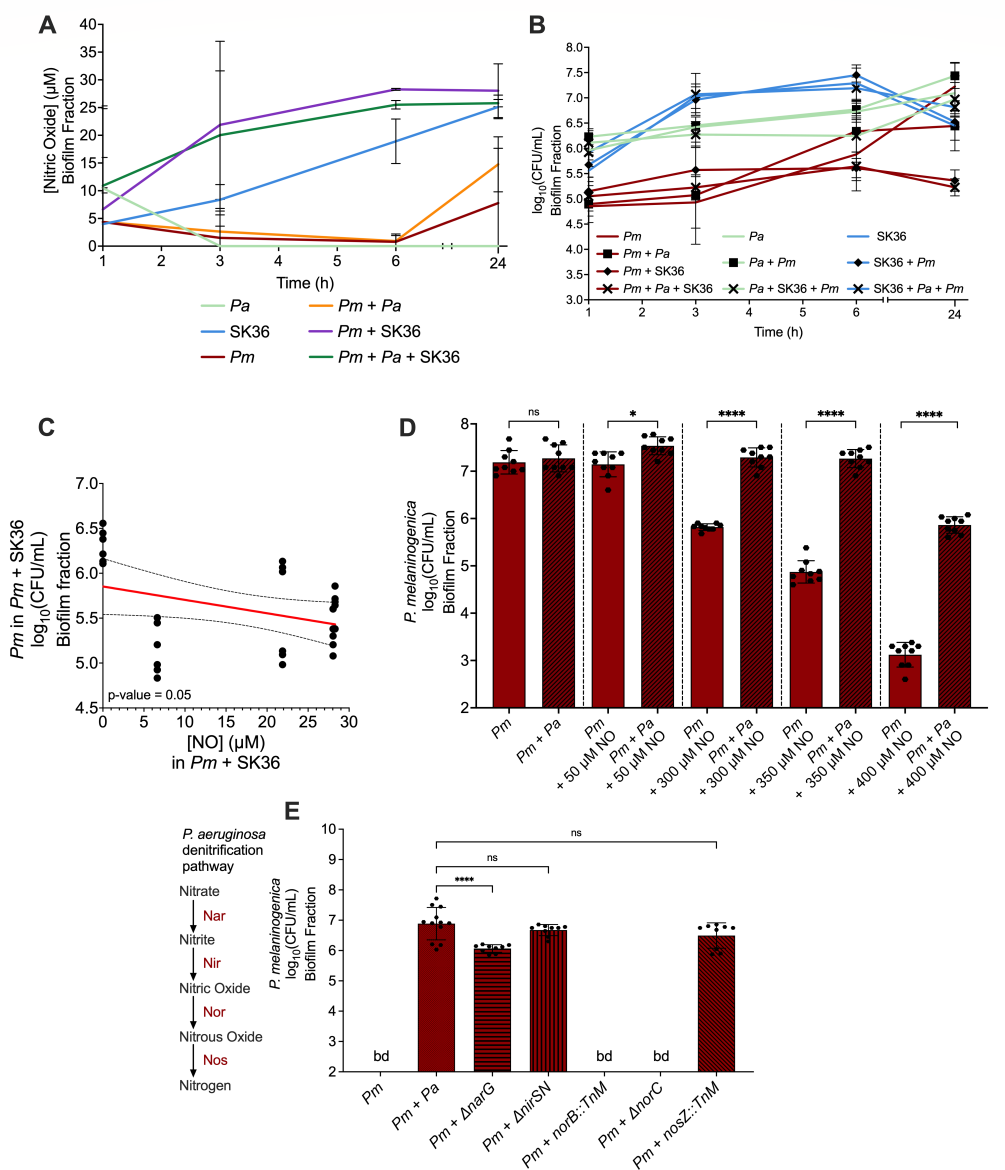
Impact of nitrosative stress on biofilm microbial interactions. (**A**) The measured concentration of nitric oxide in the biofilm fraction of culture conditions that include different combinations of *Pm*, *Pa*, and *S. sanguinis* (SK36) in modified TSBYE at 37°C over a 24 h time course with sampling times of 1, 3, 6, and 24 h. (**B**) The viable counts in the biofilm fraction from culture conditions that include different combinations of *Pm*, *Pa*, and *S. sanguinis* (SK36) in modified TSBYE at 37°C over a 24 h time course with sampling times of 1, 3, 6, and 24 h. (**C**) A scatter plot of the log_10_ (CFU/mL) of *P. melaninogenica* in co-culture with *S. sanguinis* against the concentration of NO measured in the same co-culture. A simple linear regression trend line with 95% confidence intervals was added, and the *P*-value of its slope is displayed. (**D**) The viable counts of the biofilm fraction of *Pm* when cultured in modified TSBYE under anoxic growth conditions at 37°C for 24 h and exposed to increasing concentrations of nitric oxide as either a monoculture or in co-culture with *Pa*. Statistical significance was calculated using ordinary one-way ANOVA with Tukey’s multiple comparisons test where ns = not significant, *P < 0.05, and ****P < 0.0001. Additional statistical comparisons can be found in Table S4. (**E**) The viable counts of the biofilm fraction of *Pm* when cultured in mucin-containing ASM under anoxic growth conditions at 37°C for 24 h either as a monoculture or in co-culture with wild-type *P. aeruginosa* PA14 or a collection of PA14 denitrification mutants. Statistical significance was calculated using ordinary one-way ANOVA with Tukey’s multiple comparisons test. ns = not significant and ****, *P* < 0.0001. Additional statistical comparisons can be found in Table S4.

Certain species of oral streptococci have been shown to produce nitric oxide (NO) and reactive nitrogen species (55–57). Therefore, we next explored whether *S. sanguinis* was potentially relying on such mechanisms in its interaction with *P. melaninogenica*. To that end, we first measured the concentration of NO in the biofilm fractions of the *S. sanguinis-P. melaninogenica* co-cultures over a 24 h time course. Again, due to technical reasons associated with measuring NO using a commercial kit, as stated in the Materials and Methods section, the medium used for these co-culture time course assays was modified TSBYE, rather than mucin-containing ASM. Co-cultures that included *P. aeruginosa* with either or both of *S. sanguinis* and *P. melaninogenica* were included as controls. Additionally, medium blank wells were also included to ensure that any recorded NO was not due to medium components. We report that the concentration of NO appeared to gradually increase over time to reach a value of approximately 25 µM in the biofilm fractions of the *S. sanguinis* monoculture as well as the *S. sanguinis-P. melaninogenica* pairwise culture, and the *S. sanguinis-P. melaninogenica-P. aeruginosa* triple culture (Fig. 6A). On the other hand, the *P. aeruginosa* monoculture did not appear to have measurable amounts of NO by the end of the time course (Fig. 6A).

The *P. melaninogenica* monoculture, as well as the *P. melaninogenica-P. aeruginosa* co-culture, registered NO concentrations of 7.5 µM and 14.5 µM, respectively, by the end of the time course (Fig. 6A). To better interpret these results, we analyzed the growth curves recorded during this time course. We noted that *P. melaninogenica* appeared to stay at ~10^5^ CFU/mL throughout the time course in the *P. melaninogenica-S. sanguinis* co-culture, as well as the *P. melaninogenica-S. sanguinis-P. aeruginosa* triple culture (Fig. 6B). Upon plotting the *P. melaninogenica* log_10_(CFU/mL) values in co-culture with *S. sanguinis* against the concentration of NO in the same co-culture, we observe a negatively sloped linear regression trend line with a *P*-value = 0.05, indicating a negative correlation (Fig. 6C). On the other hand, the *P. melaninogenica* monoculture, as well as the *P. melaninogenica-P. aeruginosa* co-culture, displayed steady growth over time to reach final concentrations of approximately 1.8 × 10^6^ CFU/mL and 10^7^ CFU/mL, respectively, by the endpoint of the assay. In comparison, *P. aeruginosa* and *S. sanguinis* did not seem to be affected by the different culture conditions in terms of their recorded growth. *P. aeruginosa* displayed a steady increase in CFU/mL to reach a final concentration of around 10^7^ CFU/mL across the different culture conditions (Fig. 6B). *S. sanguinis* grew to about 1.8 × 10^7^ CFU/mL by the 6 h mark, before declining to around 3 × 10^6^ CFU/mL at the endpoint across the different culture conditions (Fig. 6B). Additionally, *P. aeruginosa* appeared to no longer protect *P. melaninogenica* from *S. sanguinis* in the TSBYE medium since the growth of *P. melaninogenica* in the *P. melaninogenica-S. sanguinis-P. aeruginosa* triple culture was nearly identical to that in the *P. melaninogenica-S. sanguinis* dual culture (Fig. 6B), indicating that the protective effect of *P. aeruginosa* was limited to CF-like growth conditions.

To verify that *P. aeruginosa* can protect *P. melaninogenica* from exogenous reactive nitrogen species, we grew *P. melaninogenica* as a monoculture and in co-culture with *P. aeruginosa* in modified TSBYE and exposed these cultures to increasing concentrations of NO using the NO donor (Z)-[2-aminoethyl(2-azaniumylethyl)amino]-oxido-oxidoiminoazanium (DETA or NOC-18) (58). We observed that *P. aeruginosa* consistently protected *P. melaninogenica* from RNS (Fig. 6D). With the gradual increase in NO concentrations, the gap between the concentration of *P. melaninogenica* in monoculture compared to co-culture with *P. aeruginosa* continued to widen until reaching the highest tested concentration of exogenous NO at 400 µM, where the *P. melaninogenica* was only recoverable at 10^3^ CFU/mL in monoculture, but was detectable at 10^6^ CFU/mL in co-culture with *P. aeruginosa* (Fig. 6D). It is important to note that *P. aeruginosa* was largely unaffected by the presence of nitric oxide in its culture medium irrespective of the culture condition, except when the concentration of NO was 50 µM, where the presence of *P. melaninogenica* in co-culture resulted in significantly better *P. aeruginosa* viability (Fig. S2A), and at 400 µM, where the presence of *P. melaninogenica* in the co-culture was significantly detrimental to *P. aeruginosa* in its response to NO stress compared to monoculture (Fig. S2A). Of note, the NO concentrations used were calculated prior to the co-culture experiments by adding DETA at increasing concentrations to sterile medium under anaerobic conditions for 24 h to simulate the co-culture experiment, then measuring the concentration of NO at the end of the incubation period (Fig. S2B).

Since *P. aeruginosa* relies on denitrification for anaerobic respiration, which is a process that produces nitrates and nitric oxides, and the cultures we were testing were all under anoxic growth conditions with a nitrate source added, we wanted to investigate whether *P. aeruginosa* denitrification mutants could support the growth of *P. melaninogenica* compared to wild-type *P. aeruginosa*. Therefore, we anoxically co-cultured *P. melaninogenica* in ASM with a collection of *P. aeruginosa* nitrogen metabolism mutants that cover every enzyme involved in the denitrification process (Fig. 6E). Following incubation, we observed that *P. melaninogenica* was recoverable with *P. aeruginosa* Δ*nirS* and *nosZ*::TnM mutants to the same extent as wild-type *P. aeruginosa* at approximately 10^7^ CFU/mL (Fig. 6E). However, the detection of *P. melaninogenica* was modestly but significantly reduced by 10-fold when co-cultured with *P. aeruginosa* Δ*narG* and was completely undetectable when co-cultured with *P. aeruginosa* Δ*norC* or *norB*::TnM mutants (Fig. 6E). It is important to note that the viability of *P. aeruginosa* nitrogen metabolism mutants was not affected by the presence of *P. melaninogenica* in co-culture as their concentrations following incubation were not significantly different from their respective monocultures (Fig. S2C). Therefore, the protection that *P. aeruginosa* provides to *P. melaninogenica* against NO stress might be a function of the anaerobic respiration process that *P. aeruginosa* undergoes.

Taking our experimental data together, we conclude that *S. sanguinis* might be using a combination of both reactive nitrogen species, as well as potentially other unexplored mechanisms, to antagonize *P. melaninogenica* in co-culture. Our data are insufficient to support a direct link between hydrogen peroxide production by *S. sanguinis* and its antagonistic relationship with *P. melaninogenica*. Additionally, although *P. aeruginosa* protected and supported the growth of *P. melaninogenica* in co-culture using ASM and was also able to protect *P. melaninogenica* from reactive oxygen and nitrogen species in isolation, the benefit of culturing *P. melaninogenica* with *P. aeruginosa* was limited to the CF-like growth conditions of ASM.

## DISCUSSION

The introduction of ETI has been a landmark achievement in CF care, dramatically reducing pulmonary exacerbations and the need for lung transplants (1). However, recent studies have demonstrated that the canonical CF pathogens manage to persist in the respiratory samples retrieved from pwCF who had been on ETI for several months to a few years (9, 15, 27, 59). This discrepancy in clinical and microbiological observations prompted our interest in launching a large-scale effort to retrieve publicly available CF respiratory microbiome data from both pre- and post-ETI time periods to interrogate the observed microbial changes, including in light of microbial interactions.

Our analysis of over 4,000 respiratory samples, first stratified by ETI status, then by age group and sample type (Table S1), revealed that ETI therapy generally reduces the density of microbial co-occurrence networks and increases the number of isolated genera (Fig. 3), thus potentially disrupting the pre-ETI microbial communities. Post-ETI studies have reported an increase in CF sputum microbiome diversity (21, 59), which is an observation with an optimistic outlook because historically, low-diversity microbiomes lead to worse health outcomes (60). However, an increase in microbial diversity without community building might transition microbiomes from complex and interconnected communities to ones potentially dominated by fewer, more resilient organisms capable of thriving in the altered respiratory conditions and exerting a top-down control of their environment. For example, we observed that key pathogenic genera such as *Pseudomonas* and *Staphylococcus* were reduced in post-ETI sputum samples; however, they were the highest-ranking genera in terms of abundance and prevalence in post-ETI sinonasal samples (Fig. 1), so the upper respiratory tract can potentially act as a seeding ground for future lower respiratory tract infections. Granted, a limitation to these analyses is that most post-ETI microbiomes have so far been collected from adults who have had established CF-adapted respiratory microbiomes in different regions of their pulmonary system. Therefore, we speculate that the observed microbial response to ETI might have been influenced by former community dynamics. Regardless, analyzing the different sample types pre- and post-ETI (Fig. 1 and 3) has revealed that community models should be tailored to specific regions in the CF respiratory system because there exists heterogeneity in microbial community compositions among different regions.

In this study, we leveraged an established clinically-informed and sputum-derived CF microbial community model composed of *P. aeruginosa*, *S. aureus*, *S. sanguinis*, and *P. melaninogenica* (37) to help us understand the nature of the interaction between *S. sanguinis* and *P. melaninogenica* that emerged from our analysis of the clinical data sets. A central finding from our observations was that *S. sanguinis* antagonizes *P. melaninogenica* in co-culture (Fig. 4). This experimental finding was in line with observed changes in the sputum samples from pwCF post-ETI, where there was a sharp increase in the relative abundance of *Streptococcus*, while *Prevotella* exhibited a decline in relative abundance, mirroring that of *Pseudomonas* (Fig. 1D and G). It is important to note that while we tested multiple clinical strains to develop and validate this co-culture model (37), in these studies we focused solely on laboratory strains.

The mechanisms underlying the *S. sanguinis-P. melaninogenica* antagonism are multifaceted and appear to involve a combination of reactive nitrogen species and potentially other unexplored mechanisms. Upon measuring the concentration of NO in the different co-culture conditions, we observed that *S. sanguinis* was responsible for the increased production of NO, especially in co-culture conditions (Fig. 6A). Using TSBYE medium, which supports the growth of all organisms, the number of viable *P. melaninogenica* in co-culture with *S. sanguinis* is ~10^5^ CFU/mL throughout the time course, meanwhile the *P. melaninogenica* monoculture displayed gradual growth over time (Fig. 6A). Therefore, in co-culture with *S. sanguinis*, *P. melaninogenica* might be unable to grow due to the reactive nitrogen stress imposed by *S. sanguinis. P. aeruginosa* displayed an ability to partially shield *P. melaninogenica* from NO stress (Fig. 6D). The protective capacity of *P. aeruginosa* appears to be linked to its core metabolic processes of anaerobic denitrification, given the inability of *P. aeruginosa* ∆*narG* and ∆*norC*/*norB*::TnM mutants to support *P. melaninogenica* growth. These findings indicate that the potential build-up of nitrate and nitric oxide due to the disruption of the denitrification pathway might be detrimental to the survival of *P. melaninogenica*. One limitation of our model is that *S. sanguinis* SK36 is known to produce NO at ~25 µM (Fig. 6A), while *P. melaninogenica* is sensitive to >50 µM NO (Fig. 6D). One explanation for this discrepancy is that *P. melaninogenica* might be more sensitive to NO in the context of the peroxide that is also produced by *S. sanguinis* SK36. Additionally, our co-culture interactions were mostly tested using modified TSBYE, which is a medium that is not as representative of the CF lung nutritional environment as ASM.

Future research must continue to dissect these polymicrobial dynamics in the post-ETI era. The analysis presented here may generate hypotheses regarding the changing nature of microbial interactions that can be tested *in vitro*. Understanding how the altered nutritional and inflammatory landscape of the ETI-treated lung impacts microbial community structure will be crucial for developing next-generation therapies that look beyond a single pathogen and instead aim to modulate or remodel the entire microbial ecosystem for the benefit of the host.

## MATERIALS AND METHODS

### CF lung microbiome analysis

The data sets used to analyze the CF respiratory microbiome were retrieved from studies with publicly available sequencing data (3–12, 14, 15, 18, 19, 21, 23–25). The pre-ETI data sets were downloaded from the Dartmouth RESPIRE Database (https://respire.dartmouth.edu/), while the post-ETI data sets were downloaded from NCBI BioProject using the Google Cloud service BigQuery. Metadata relating the bacterial counts from each sample of each study to its respective anonymized patient demographic information was manually retrieved, either from the supplemental material provided by the study or by contacting the authors directly. The resulting curated spreadsheets containing bacterial counts and genera, sampling sources, ETI status, and patient age groups were loaded onto RStudio for downstream analyses. Most of the post-ETI studies were longitudinal studies; therefore, only data from the last sample retrieved from each patient were included in the analyzed data set, thus providing the most recent representation of the CF respiratory microbiome that was exposed to ETI for the longest time. Data set characteristics can be found in Table S1.

Prevalence and relative abundances (Fig. 1 and 2) were determined using a previously established source code (37) that was modified to fit our data sets. Briefly, relative abundances were calculated by normalizing each sample’s genus-level read counts to its total reads. Prevalence was computed as the fraction of samples in which each genus was detected, and mean relative abundance was averaged across samples. Only genera present in more than 20% of samples were retained, and mean abundances were log-transformed. The most abundant genera were selected for plotting in each data set. Plots were generated using ggplot2 (61), labeled using ggrepel (62), and combined into multi-panel figures using patchwork (63). The dplyr package (64) was used for data manipulation.

Microbial correlation network analysis (Fig. 3 and 4) was performed using the igraph (65–67), Hmisc (68), and corrplot (69) packages to explore co-occurrence patterns among the most abundant genera across multiple sample types and clinical groups before and after ETI treatment. Pairwise Spearman correlations were computed using relative abundance data. Spearman correlations that were strong (|r_s_| > 0.5) and statistically significant (*P* < 0.05) (70) were retained and visualized via heatmaps. Microbial co-occurrence networks were constructed to be undirected and weighted, with nodes representing genera and edges denoting positive (orange) or negative (purple) correlations. Node sizes were scaled by degree centrality, and edge widths reflected correlation strength. Network metrics such as degree and edge density were calculated, and community detection was performed using the Louvain algorithm (71) to identify clusters of co-associated genera. The R code used to perform all analyses can be retrieved from https://github.com/bassamhafi3/cflungmicrobiome.

### Bacterial strains and culture conditions

*P. melaninogenica* ATCC 25845 (72), *S. sanguinis* SK36 (73), *P. aeruginosa* PA14 (74), and *S. aureus* Newman (75) were included in this study and cultured as previously described (37). The strains used in this study and the plasmids used to build mutant strains are listed in Table S3. Briefly, *P. melaninogenica* was cultured overnight at 37°C in either a Thermo Fisher Scientific AnaeroPack anaerobic box with a BD GasPak anaerobe sachet, or a Coy Laboratory anaerobic chamber, using modified TSBYE (Mod. TSBYE) composed of tryptic soy broth with 0.5% yeast extract, 500 μg/mL L-cysteine, 5 μg/mL hemin, and 1 μg/mL menadione. *S. sanguinis* was cultured overnight at 37°C + 5% CO_2_ in Todd-Hewitt broth with 0.5% yeast extract. *P. aeruginosa* and *S. aureus* were cultured overnight at 37°C in lysogeny broth with shaking.

### Bacterial co-culture assays

All co-culture experiments were conducted following a previously established protocol (37, 40, 76) with modifications that tailor certain steps in the protocol to the different conditions that were tested. In general, overnight liquid cultures of the tested strains were collected and centrifuged into pellets that were then washed with 1× PBS and resuspended in fresh culture media. The bacterial suspensions were then normalized to an optical density OD_600_ = 0.05. To test monocultures, the OD-normalized suspensions were directly dispensed into a 96-well plate containing media for a final OD_600_ = 0.01. To test mixed cultures, the OD-normalized suspensions were combined so that each strain achieved an OD_600_ = 0.01, and then the mixtures were dispensed into a 96-well plate. The 96-well culture plates were then incubated at 37°C for 24 h (unless otherwise noted) in either a Thermo Fisher Scientific AnaeroPack anaerobic box with a BD GasPak anaerobe sachet, or in a Coy Laboratory anaerobic chamber. Following incubation, the planktonic fractions of the cultures were separated from the biofilm fractions, and 50 µL of 1× PBS was added to each culture well of the 96-well plates. The biofilm fractions were then detached using a 96-pin replicator and serially diluted by 10-fold increments to 10^−7^. The entire dilution series was then spotted onto selective media and incubated overnight under the appropriate culture conditions for each organism. The resulting CFU of the biofilm fraction were enumerated, and the concentrations of each organism were determined by calculating their CFU/mL.

The media used to resuspend the cell pellets and grow the mono- and mixed cultures were either artificial sputum medium (37, 39, 76) or modified TSBYE, as indicated. The selective medium used to spot the dilution series was *Prevotella* selective agar, composed of blood agar supplemented with 500 μg/mL cysteine, 100 μg/mL kanamycin, 7.5 μg/mL vancomycin, 5 μg/mL polymyxin B, 5 μg/mL hemin, and 1 μg/mL menadione. *Streptococcus* selective agar, made of blood agar supplemented with 10 μg/mL polymyxin B and 10 μg/mL oxolinic acid. *Pseudomonas* isolation agar was used to isolate *P. aeruginosa*, and mannitol salt agar to isolate *S. aureus*.

### Time course co-culture assays

The time course experiments (Fig. 5C and 6B) followed the same protocol as the 24 h co-culture experiments described above; however, several 96-well plates, corresponding to the number of time points to be measured, were initially inoculated at the same time and were then processed in sequence at their corresponding time points.

### Hydrogen peroxide measurement

Hydrogen peroxide measurements were performed on the biofilm fraction of the cultures following the detachment step as described in the co-culture experimental procedure above; however, modified TSBYE, rather than ASM, was used as the culture medium because the latter contains mucin, which has insoluble particulates that can be autofluorescent and would interfere with plate reader fluorescence measurements. The concentrations of H_2_O_2_ were measured using the Enzo Life Sciences red hydrogen peroxide assay kit (Cat. # ENZ-51004) according to the manufacturer’s instructions. In a 96-well plate, 50 µL of the H_2_O_2_ reaction mixture, composed of peroxidase and the red peroxidase substrate, was mixed with 50 µL of the biofilm fraction of each culture condition in triplicate. In parallel, hydrogen peroxide standards were prepared with 50 µL H_2_O_2_ reaction mixture and known decreasing concentrations of hydrogen peroxide starting at 3 µM ⟶ 0.01 µM in 1:3 dilution increments. Finally, blank wells of the H_2_O_2_ reaction mixture with 1× PBS were also prepared. The 96-well plate was then incubated in the dark at room temperature for 15 min. Following incubation, fluorescence was measured at Ex/Em = 540/590 nm using a microplate reader. A standard curve was generated, and the concentration of hydrogen peroxide in each test well was measured against the standard curve.

### Nitric oxide measurement

Nitric oxide measurements were performed on the biofilm fraction of the cultures following the detachment step as described in the co-culture experimental procedure above; however, modified TSBYE, rather than ASM, was used as the culture medium because the latter contains mucin, which has insoluble particulates that can interfere with plate reader optical density measurements. The concentrations of NO were measured using the Enzo Life Sciences nitric oxide total detection kit (Cat. # ADI-917-020). The manufacturer’s instructions describe a procedure that measures NO indirectly from the amount of nitrate and nitrite found in the medium. In a 96-well plate, 50 µL of the reaction buffer, which was prepared by diluting a concentrate that came with the kit, was mixed with 50 µL of the biofilm fraction of each culture condition in triplicate. In parallel, nitrate standards were prepared by diluting a 1,000 µmol/L concentrate with reaction mixture in a serial dilution series to generate standards with known concentrations starting at 100 µmol/L ⟶ 3.125 µmol/L. To both standard and test wells, 25 µL of the NADH reagent and 25 µL of the nitrate reductase enzyme were added. Finally, 200 µL blank wells of the reaction mixture with 1× PBS were also prepared. The 96-well plate was then incubated at 37°C for 30 min. Following incubation, 50 µL of Griess reagent I and 50 µL of Griess reagent II were added to each of the standard and test wells only, and the plate was incubated at room temperature for 10 min. The optical density of each well was then measured at 550 nm using a microplate reader. A standard curve was generated, and the concentration of NO in each test well was measured against the standard curve.

### Statistical analysis

Ordinary one-way ANOVA was used with GraphPad Prism 10 to determine statistical significance of the co-culture experimental results as indicated in the figure legends. Spearman correlation analyses were performed using R (v.4.5.1) with cutoff thresholds of |*r*_s_| > 0.5 and *P*-value <0.05 to analyze the pre- and post-ETI microbiome data sets, which retain associations that may be biologically meaningful, and reduce noise in the resulting network (70). Simple linear regression was used with GraphPad Prism 10 to analyze the relationship between *P. melaninogenica* enumerated CFU in co-culture with *S. sanguinis* and the concentrations of either hydrogen peroxide or nitric oxide in the co-cultures. Additional statistical comparisons can be found in Table S4.

## SUPPLEMENTAL MATERIAL

Supplemental figures jb.00005-26-s0001.pdf Figures S1 and S2.

Table S1 jb.00005-26-s0002.pdf CF respiratory microbiome dataset characteristics for both pre- and post-ETI samples.

Table S2 jb.00005-26-s0003.xlsx Spearman correlation matrices of the top 15 genera by relative abundance levels.

Table S3 jb.00005-26-s0004.pdf The list of bacterial strains and plasmid deletion vectors used in this study.

Table S4 jb.00005-26-s0005.pdf Ordinary one-way analysis of variance (ANOVA) with Tukey's multiple comparisons test for Figures 4-6.

ASM does not own the copyrights to Supplemental Material that may be linked to, or accessed through, an article. The authors have granted ASM a non-exclusive, world-wide license to publish the Supplemental Material files. Please contact the corresponding author directly for reuse.
