# Supplementary material for: Streptococcus sanguinis antagonizes Prevotella melaninogenica in the context of the cystic fibrosis respiratory microbiome
Source: J Bacteriol. 2026 Feb 27;208(3):e00005-26. doi: 10.1128/jb.00005-26 (PMC13001228; doi:10.1128/jb.00005-26)
Supplement: Supplemental figures — Figures S1 and S2. [file jb.00005-26-s0001.pdf]

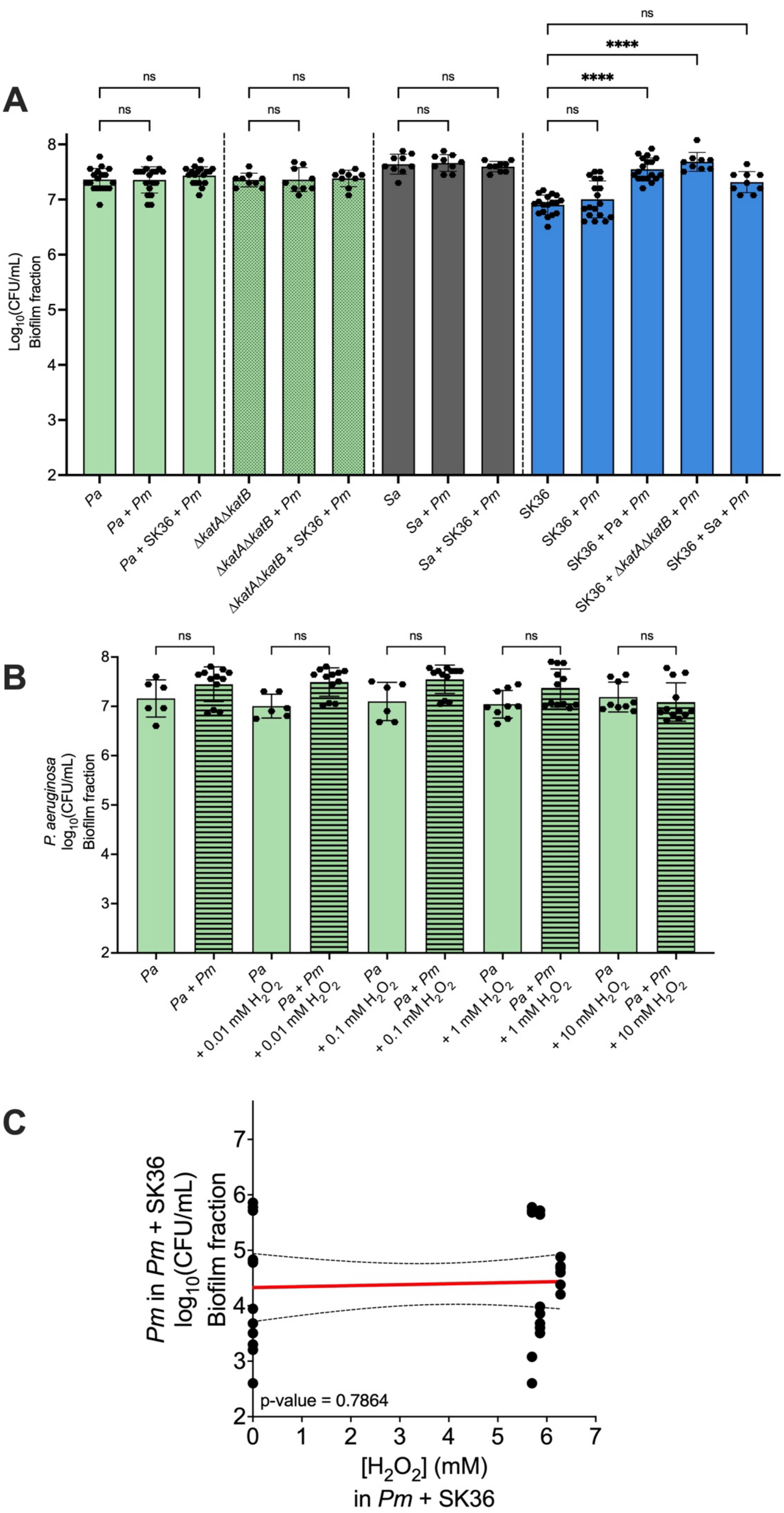

A
ns
ns
****
****
Log10(CFU/mL)
Biofilm fraction
Pa
Pa + Pm
Pa + SK36 + Pm
ΔkatAΔkatB
ΔkatAΔkatB + Pm
ΔkatAΔkatB + SK36 + Pm
Sa
Sa + Pm
Sa + SK36 + Pm
SK36
SK36 + Pm
SK36 + Pa + Pm
SK36 + ΔkatAΔkatB + Pm
SK36 + Sa + Pm
B
P. aeruginosa
log10(CFU/mL)
Biofilm fraction
Pa + 0.01 mM H2O2
Pa + Pm + 0.01 mM H2O2
Pa + 0.1 mM H2O2
Pa + Pm + 0.1 mM H2O2
Pa + 1 mM H2O2
Pa + Pm + 1 mM H2O2
Pa + 10 mM H2O2
Pa + Pm + 10 mM H2O2
C
Pm in Pm + SK36
log10(CFU/mL)
Biofilm fraction
p-value = 0.7864
[H2O2] (mM)
in Pm + SK36

Supplementary Figure S1. (A) All cultures were performed using mucin-containing ASM under anoxic growth conditions at 37ºC for 24 hours. The viable counts of the biofilm fractions of the co-cultures are plotted, showing the viable counts of *P. aeruginosa* PA14 (*Pa*), *P. aeruginosa* PA14 Δ*katA*Δ*katB* (Δ*katA*Δ*katB*), *S. sanguinis* SK36 (S36), and *S. aureus* Newman (*Sa*) in different co-culture combinations with each other and with *P. melaninogenica* ATCC 25845 (*Pm*). Statistical significance was calculated using ordinary one-way analysis of variance (ANOVA) with Tukey's multiple comparisons test. ns = not significant and ****, $p < 0.0001$. (B) All cultures were performed using modified TSBYE under anoxic growth conditions at 37ºC for 24 hours. Displaying the viable counts of the biofilm fraction of *P. aeruginosa* PA14 (*Pa*) when exposed to increasing concentrations of H_2O_2 as either a monoculture or in co-culture with *P. melaninogenica* (*Pm*). Statistical significance was calculated using ordinary one-way analysis of variance (ANOVA) with Tukey's multiple comparisons test. ns = not significant. (C) A scatter plot of the $\log_{10}$(CFU/mL) of *P. melaninogenica* in co-culture with *S. sanguinis* against the concentration of H_2O_2 measured in the same co-culture. A simple linear regression trend line with 95% confidence interval was added and the p-value of its slope is displayed.

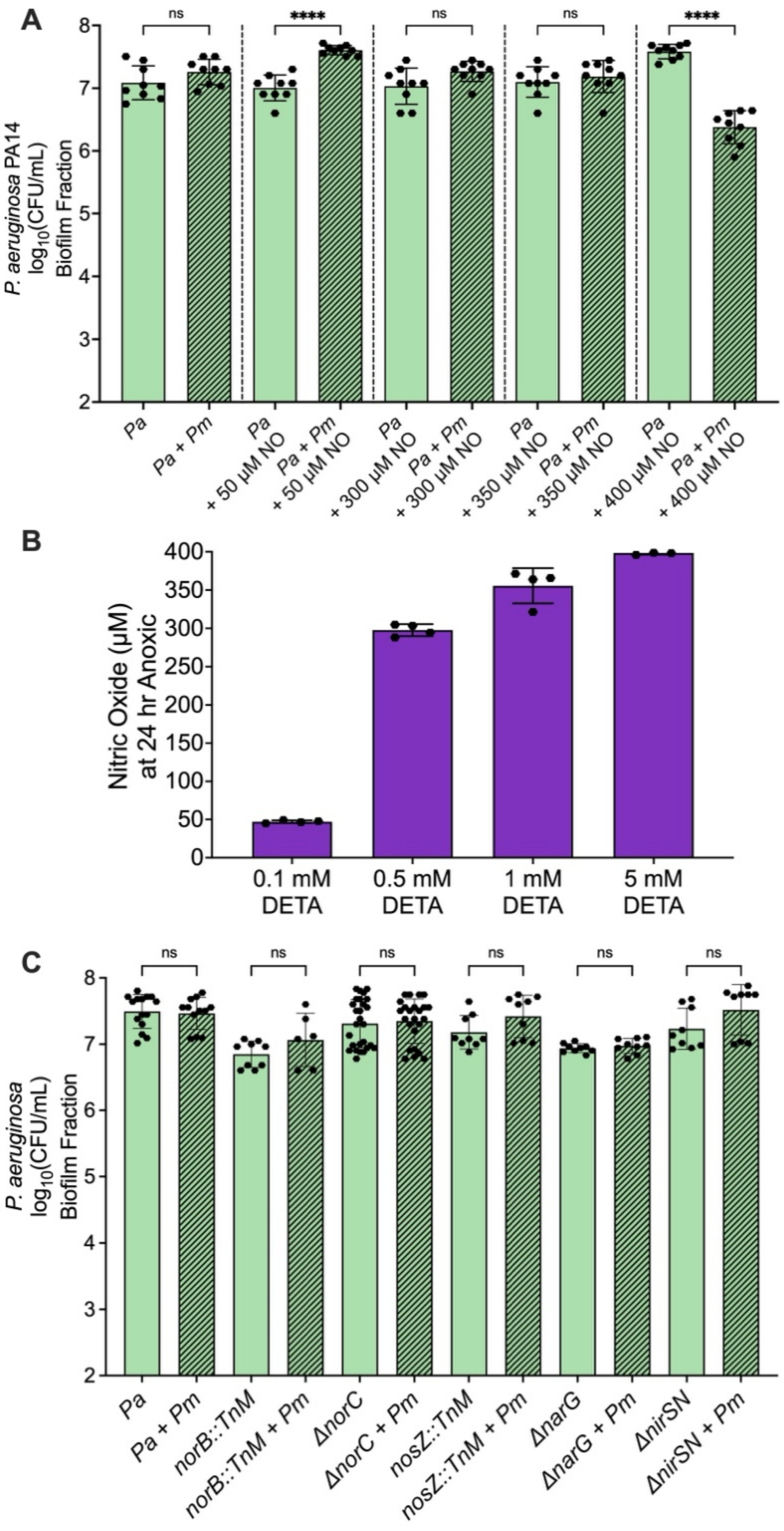
A
B
C
P. aeruginosa PA14 log10(CFU/mL) Biofilm Fraction
ns
****
Pa
Pa + Pm
Pa + 50 µM NO
Pa + Pm + 50 µM NO
Pa + 300 µM NO
Pa + Pm + 300 µM NO
Pa + 350 µM NO
Pa + Pm + 350 µM NO
Pa + 400 µM NO
Pa + Pm + 400 µM NO
Nitric Oxide (µM) at 24 hr Anoxic
0.1 mM DETA
0.5 mM DETA
1 mM DETA
5 mM DETA
P. aeruginosa log10(CFU/mL) Biofilm Fraction
norB::TnM
norB::TnM + Pm
ΔnorC
ΔnorC + Pm
nosZ::TnM
nosZ::TnM + Pm
ΔnarG
ΔnarG + Pm
ΔnirSN
ΔnirSN + Pm

Supplementary Figure S2. (A) All cultures were performed using modified TSBYE under anoxic growth conditions at 37ºC for 24 hours. Displaying the viable counts of the biofilm fraction of *P. aeruginosa* PA14 (*Pa*) when exposed to increasing concentrations of nitric oxide (NO) as either a monoculture or in co-culture with *P. melaninogenica* (*Pm*). Statistical significance was calculated using ordinary one-way analysis of variance (ANOVA) with Tukey's multiple comparisons test. ns = not significant and ****, $p < 0.0001$. (B) The concentration of nitric oxide measured in the medium as a function of the concentrations of NO donor DETA that was added at the start of the experiment. (C) All cultures were performed using mucin-containing ASM under anoxic growth conditions at 37ºC for 24 hours. The viable counts of the biofilm fractions of the co-cultures are plotted, showing the viable counts of *P. aeruginosa* PA14 (*Pa*), as well as the indicated *P. aeruginosa* denitrification mutants in different co-culture combinations with *P. melaninogenica* ATCC 25845 (*Pm*). Statistical significance was calculated using ordinary one-way analysis of variance (ANOVA) with Tukey's multiple comparisons test. ns = not significant
