# Supplementary material for: Streptococcus sanguinis antagonizes Prevotella melaninogenica in the context of the cystic fibrosis respiratory microbiome
Source: J Bacteriol. 2026 Feb 27;208(3):e00005-26. doi: 10.1128/jb.00005-26 (PMC13001228; doi:10.1128/jb.00005-26)
Supplement: Table S1 — CF respiratory microbiome dataset characteristics for both pre- and post-ETI samples. [file jb.00005-26-s0002.pdf]

Supplementary Table S1: CF respiratory microbiome dataset characteristics for both pre- and post-ETI samples.

Treatment Status	Grouping		# of Samples
Pre-ETI (n = 3897)	Age	Adult	741
		Sputum	612
		Oropharynx	63
		Bronchoalveolar Lavage	25
		Pediatric	2365
		Sputum	215
		Oropharynx	1652
		Bronchoalveolar Lavage	408
		Unclassifiable	791
	Sample Type	Sputum	1500
		Bronchoalveolar Lavage	430
		Oropharynx	1746
		Others[a]	221
Post-ETI (n = 335)	Age	Adult	180
		Sputum	71
		Oropharynx	25
		Sinonasal	84
		Pediatric	8
		Sinonasal	8
		Unclassifiable	147
	Sample Type	Sputum	218
		Oropharynx	25
		Sinonasal	92

[a]Others: Nasal lavage, saliva, protected brush, and unlabeled sample types.
