# Supplementary material for: Streptococcus sanguinis antagonizes Prevotella melaninogenica in the context of the cystic fibrosis respiratory microbiome
Source: J Bacteriol. 2026 Feb 27;208(3):e00005-26. doi: 10.1128/jb.00005-26 (PMC13001228; doi:10.1128/jb.00005-26)
Supplement: Table S3 — The list of bacterial strains and plasmid deletion vectors used in this study. [file jb.00005-26-s0004.pdf]

Supplementary Table S3. The list of bacterial strains and plasmid deletion vectors used in this study.

**Strain**	**Genotype**	**Reference/Source**
P. melaninogenica ATCC 25845	Wildtype	(1)
S. sanguinis SK36	Wildtype	(2)
S. aureus Newman	Wildtype	(3)
P. aeruginosa PA14	Wildtype	(4)
P. aeruginosa PA14	Δ*katA*Δ*katB*	(5)
P. aeruginosa PA14	Δ*narG*	This study
P. aeruginosa PA14	Δ*nirSN*	This study
P. aeruginosa PA14	Δ*norC*	This study
P. aeruginosa PA14	*norB*::TnM	(6)
P. aeruginosa PA14	*nosZ*::TnM	(6)
S. sanguinis SK36	Δ*spxB*	(7)

**Plasmid**	**Reference/Source**
pMQ30::*narG*-KO	This study
pMQ30::*nirSN*-KO	This study
pMQ30::*norC*-KO	This study

References

1. Shah HN, Collins DM. 1990. *Prevotella*, a new genus to include *Bacteroides melaninogenicus* and related species formerly classified in the genus *Bacteroides*. International Journal of Systematic and Evolutionary Microbiology 40:205–208.
2. Kilian M, Holmgren K. 1981. Ecology and nature of immunoglobulin A1 protease-producing Streptococci in the human oral cavity and pharynx. Infection and Immunity 31:868–873.
3. Duthie ES. 1952. Variation in the antigenic composition of staphylococcal coagulase. Microbiology 7:320–326.
4. Rahme LG, Stevens EJ, Wolfort SF, Shao J, Tompkins RG, Ausubel FM. 1995. Common virulence factors for bacterial pathogenicity in plants and animals. Science 268:1899–1902.
5. Scott JE, Li K, Filkins LM, Zhu B, Kuchma SL, Schwartzman JD, O'Toole GA. 2019. *Pseudomonas aeruginosa* can inhibit growth of streptococcal species via siderophore production. Journal of Bacteriology 201:e00014-19.
6. Liberati NT, Urbach JM, Miyata S, Lee DG, Drenkard E, Wu G, Villanueva J, Wei T, Ausubel FM. 2006. An ordered, nonredundant library of *Pseudomonas aeruginosa* strain PA14 transposon insertion mutants. Proceedings of the National Academy of Sciences of the United States of America 103:2833–2838.
7. Chen L, Ge X, Xu P. 2015. Identifying essential Streptococcus sanguinis genes using genome-wide deletion mutation. Methods Mol Biol 1279:15–23
