# Supplementary material for: Streptococcus sanguinis antagonizes Prevotella melaninogenica in the context of the cystic fibrosis respiratory microbiome
Source: J Bacteriol. 2026 Feb 27;208(3):e00005-26. doi: 10.1128/jb.00005-26 (PMC13001228; doi:10.1128/jb.00005-26)
Supplement: Table S4 — Ordinary one-way analysis of variance (ANOVA) with Tukey's multiple comparisons test for Figures 4-6. [file jb.00005-26-s0005.pdf]

Supplementary Table S4: Ordinary one-way analysis of variance (ANOVA) with Tukey's multiple comparisons test for Figures 4-6:

Figure	Panel	Comparison	P-value
4	A	Pm + Pa vs. Pm + Sa	<0.0001
		Pm + Pa vs. Pm + Pa + SK36	<0.0001
		Pm + Sa vs. Pm + Pa + SK36	>0.9999
	B	SK36 vs. SK36 + Pm	0.9984
		SK36 vs. Pm	<0.0001
		SK36 vs. Pm + SK36	<0.0001
		SK36 + Pm vs. Pm	<0.0001
		SK36 + Pm vs. Pm + SK36	<0.0001
		Pm vs. Pm + SK36	<0.0001
	C	Pm vs. Pm TSBYE 3:1 SK36 supe	<0.0001
		Pm vs. Pm TSBYE 1:1 SK36 supe	<0.0001
		Pm vs. Pm TSBYE 1:3 SK36 supe	<0.0001
		Pm vs. Pm + SK36	<0.0001
		Pm TSBYE 3:1 SK36 supe vs. Pm TSBYE 1:1 SK36 supe	0.39
		Pm TSBYE 3:1 SK36 supe vs. Pm TSBYE 1:3 SK36 supe	0.9202
		Pm TSBYE 3:1 SK36 supe vs. Pm + SK36	0.9944
		Pm TSBYE 1:1 SK36 supe vs. Pm TSBYE 1:3 SK36 supe	0.9959
		Pm TSBYE 1:1 SK36 supe vs. Pm + SK36	0.9319
		Pm TSBYE 1:3 SK36 supe vs. Pm + SK36	>0.9999
5	A	Pm vs. Pm + Pa	0.9498
		Pm vs. Pm + 0.01 mM H2O2	>0.9999
		Pm vs. Pm + Pa + 0.01 mM H2O2	0.9901
		Pm vs. Pm + 0.1 mM H2O2	0.8987
		Pm vs. Pm + Pa + 0.1 mM H2O2	0.9068
		Pm vs. Pm + 1 mM H2O2	<0.0001
		Pm vs. Pm + Pa + 1 mM H2O2	0.1317
		Pm vs. Pm + 10 mM H2O2	<0.0001
		Pm vs. Pm + Pa + 10 mM H2O2	<0.0001
		Pm vs. Pm + 100 mM H2O2	<0.0001
		Pm vs. Pm + Pa + 100 mM H2O2	<0.0001
		Pm + Pa vs. Pm + 0.01 mM H2O2	0.9987
		Pm + Pa vs. Pm + Pa + 0.01 mM H2O2	>0.9999
		Pm + Pa vs. Pm + 0.1 mM H2O2	0.0065
		Pm + Pa vs. Pm + Pa + 0.1 mM H2O2	>0.9999

		Pm + Pa vs. Pm + 1 mM H2O2	<0.0001
		Pm + Pa vs. Pm + Pa + 1 mM H2O2	0.9912
		Pm + Pa vs. Pm + 10 mM H2O2	<0.0001
		Pm + Pa vs. Pm + Pa + 10 mM H2O2	<0.0001
		Pm + Pa vs. Pm + 100 mM H2O2	<0.0001
		Pm + Pa vs. Pm + Pa + 100 mM H2O2	<0.0001
		Pm + 0.01 mM H2O2 vs. Pm + Pa + 0.01 mM H2O2	>0.9999
		Pm + 0.01 mM H2O2 vs. Pm + 0.1 mM H2O2	0.774
		Pm + 0.01 mM H2O2 vs. Pm + Pa + 0.1 mM H2O2	0.996
		Pm + 0.01 mM H2O2 vs. Pm + 1 mM H2O2	<0.0001
		Pm + 0.01 mM H2O2 vs. Pm + Pa + 1 mM H2O2	0.4668
		Pm + 0.01 mM H2O2 vs. Pm + 10 mM H2O2	<0.0001
		Pm + 0.01 mM H2O2 vs. Pm + Pa + 10 mM H2O2	<0.0001
		Pm + 0.01 mM H2O2 vs. Pm + 100 mM H2O2	<0.0001
		Pm + 0.01 mM H2O2 vs. Pm + Pa + 100 mM H2O2	<0.0001
		Pm + Pa + 0.01 mM H2O2 vs. Pm + 0.1 mM H2O2	0.0163
		Pm + Pa + 0.01 mM H2O2 vs. Pm + Pa + 0.1 mM H2O2	>0.9999
		Pm + Pa + 0.01 mM H2O2 vs. Pm + 1 mM H2O2	<0.0001
		Pm + Pa + 0.01 mM H2O2 vs. Pm + Pa + 1 mM H2O2	0.9381
		Pm + Pa + 0.01 mM H2O2 vs. Pm + 10 mM H2O2	<0.0001
		Pm + Pa + 0.01 mM H2O2 vs. Pm + Pa + 10 mM H2O2	<0.0001
		Pm + Pa + 0.01 mM H2O2 vs. Pm + 100 mM H2O2	<0.0001
		Pm + Pa + 0.01 mM H2O2 vs. Pm + Pa + 100 mM H2O2	<0.0001
		Pm + 0.1 mM H2O2 vs. Pm + Pa + 0.1 mM H2O2	0.004
		Pm + 0.1 mM H2O2 vs. Pm + 1 mM H2O2	<0.0001
		Pm + 0.1 mM H2O2 vs. Pm + Pa + 1 mM H2O2	<0.0001
		Pm + 0.1 mM H2O2 vs. Pm + 10 mM H2O2	<0.0001
		Pm + 0.1 mM H2O2 vs. Pm + Pa + 10 mM H2O2	<0.0001
		Pm + 0.1 mM H2O2 vs. Pm + 100 mM H2O2	<0.0001
		Pm + 0.1 mM H2O2 vs. Pm + Pa + 100 mM H2O2	<0.0001
		Pm + Pa + 0.1 mM H2O2 vs. Pm + 1 mM H2O2	<0.0001
		Pm + Pa + 0.1 mM H2O2 vs. Pm + Pa + 1 mM H2O2	0.9977
		Pm + Pa + 0.1 mM H2O2 vs. Pm + 10 mM H2O2	<0.0001
		Pm + Pa + 0.1 mM H2O2 vs. Pm + Pa + 10 mM H2O2	<0.0001
		Pm + Pa + 0.1 mM H2O2 vs. Pm + 100 mM H2O2	<0.0001
		Pm + Pa + 0.1 mM H2O2 vs. Pm + Pa + 100 mM H2O2	<0.0001
		Pm + 1 mM H2O2 vs. Pm + Pa + 1 mM H2O2	<0.0001
		Pm + 1 mM H2O2 vs. Pm + 10 mM H2O2	>0.9999
		Pm + 1 mM H2O2 vs. Pm + Pa + 10 mM H2O2	<0.0001

		Pm + 1 mM H2O2 vs. Pm + 100 mM H2O2	>0.9999
		Pm + 1 mM H2O2 vs. Pm + Pa + 100 mM H2O2	<0.0001
		Pm + Pa + 1 mM H2O2 vs. Pm + 10 mM H2O2	<0.0001
		Pm + Pa + 1 mM H2O2 vs. Pm + Pa + 10 mM H2O2	0.0103
		Pm + Pa + 1 mM H2O2 vs. Pm + 100 mM H2O2	<0.0001
		Pm + Pa + 1 mM H2O2 vs. Pm + Pa + 100 mM H2O2	<0.0001
		Pm + 10 mM H2O2 vs. Pm + Pa + 10 mM H2O2	<0.0001
		Pm + 10 mM H2O2 vs. Pm + 100 mM H2O2	>0.9999
		Pm + 10 mM H2O2 vs. Pm + Pa + 100 mM H2O2	<0.0001
		Pm + Pa + 10 mM H2O2 vs. Pm + 100 mM H2O2	<0.0001
		Pm + Pa + 10 mM H2O2 vs. Pm + Pa + 100 mM H2O2	<0.0001
		Pm + 100 mM H2O2 vs. Pm + Pa + 100 mM H2O2	<0.0001
	D	SK36 vs. SK36 + Pm	>0.9999
		SK36 vs. SK36 ΔspxB	>0.9999
		SK36 vs. SK36 ΔspxB + Pm	>0.9999
		SK36 vs. Pm	0.8536
		SK36 vs. Pm + SK36 WT	0.0003
		SK36 vs. Pm + SK36 ΔspxB	0.4736
		SK36 + Pm vs. SK36 ΔspxB	>0.9999
		SK36 + Pm vs. SK36 ΔspxB + Pm	>0.9999
		SK36 + Pm vs. Pm	0.9986
		SK36 + Pm vs. Pm + SK36 WT	<0.0001
		SK36 + Pm vs. Pm + SK36 ΔspxB	0.1122
		SK36 ΔspxB vs. SK36 ΔspxB + Pm	>0.9999
		SK36 ΔspxB vs. Pm	0.5049
		SK36 ΔspxB vs. Pm + SK36 WT	0.3264
		SK36 ΔspxB vs. Pm + SK36 ΔspxB	0.9997
		SK36 ΔspxB + Pm vs. Pm	0.9307
		SK36 ΔspxB + Pm vs. Pm + SK36 WT	0.0577
		SK36 ΔspxB + Pm vs. Pm + SK36 ΔspxB	0.9532
		Pm vs. Pm + SK36 WT	<0.0001
		Pm vs. Pm + SK36 ΔspxB	0.0001
		Pm + SK36 WT vs. Pm + SK36 ΔspxB	0.9996
	E	Pm + Pa vs. Pm + ΔkatAΔkatB	0.9848
		Pm + Pa vs. Pm + Pa + SK36	<0.0001
		Pm + Pa vs. Pm + ΔkatAΔkatB + SK36	<0.0001
		Pm + ΔkatAΔkatB vs. Pm + Pa + SK36	<0.0001
		Pm + ΔkatAΔkatB vs. Pm + ΔkatAΔkatB + SK36	<0.0001
		Pm + Pa + SK36 vs. Pm + ΔkatAΔkatB + SK36	0.9991

6	D	Pm vs. Pm + Pa	>0.9999
		Pm vs. Pm + 400 μM NO	<0.0001
		Pm vs. Pm + Pa + 400 μM NO	<0.0001
		Pm vs. Pm + 350 μM NO	<0.0001
		Pm vs. Pm + Pa + 350 μM NO	>0.9999
		Pm vs. Pm + 300 μM NO	<0.0001
		Pm vs. Pm + Pa + 300 μM NO	>0.9999
		Pm vs. Pm + 50 μM NO	>0.9999
		Pm vs. Pm + Pa + 50 μM NO	0.0986
		Pm + Pa vs. Pm + 400 μM NO	<0.0001
		Pm + Pa vs. Pm + Pa + 400 μM NO	<0.0001
		Pm + Pa vs. Pm + 350 μM NO	<0.0001
		Pm + Pa vs. Pm + Pa + 350 μM NO	>0.9999
		Pm + Pa vs. Pm + 300 μM NO	<0.0001
		Pm + Pa vs. Pm + Pa + 300 μM NO	>0.9999
		Pm + Pa vs. Pm + 50 μM NO	0.9996
		Pm + Pa vs. Pm + Pa + 50 μM NO	0.53
		Pm + 400 μM NO vs. Pm + Pa + 400 μM NO	<0.0001
		Pm + 400 μM NO vs. Pm + 350 μM NO	<0.0001
		Pm + 400 μM NO vs. Pm + Pa + 350 μM NO	<0.0001
		Pm + 400 μM NO vs. Pm + 300 μM NO	<0.0001
		Pm + 400 μM NO vs. Pm + Pa + 300 μM NO	<0.0001
		Pm + 400 μM NO vs. Pm + 50 μM NO	<0.0001
		Pm + 400 μM NO vs. Pm + Pa + 50 μM NO	<0.0001
		Pm + Pa + 400 μM NO vs. Pm + 350 μM NO	<0.0001
		Pm + Pa + 400 μM NO vs. Pm + Pa + 350 μM NO	<0.0001
		Pm + Pa + 400 μM NO vs. Pm + 300 μM NO	>0.9999
		Pm + Pa + 400 μM NO vs. Pm + Pa + 300 μM NO	<0.0001
		Pm + Pa + 400 μM NO vs. Pm + 50 μM NO	<0.0001
		Pm + Pa + 400 μM NO vs. Pm + Pa + 50 μM NO	<0.0001
		Pm + 350 μM NO vs. Pm + Pa + 350 μM NO	<0.0001
		Pm + 350 μM NO vs. Pm + 300 μM NO	<0.0001
		Pm + 350 μM NO vs. Pm + Pa + 300 μM NO	<0.0001
		Pm + 350 μM NO vs. Pm + 50 μM NO	<0.0001
		Pm + 350 μM NO vs. Pm + Pa + 50 μM NO	<0.0001
		Pm + Pa + 350 μM NO vs. Pm + 300 μM NO	<0.0001
		Pm + Pa + 350 μM NO vs. Pm + Pa + 300 μM NO	>0.9999
		Pm + Pa + 350 μM NO vs. Pm + 50 μM NO	0.9998
		Pm + Pa + 350 μM NO vs. Pm + Pa + 50 μM NO	0.5013

		Pm + 300 μM NO vs. Pm + Pa + 300 μM NO	<0.0001
		Pm + 300 μM NO vs. Pm + 50 μM NO	<0.0001
		Pm + 300 μM NO vs. Pm + Pa + 50 μM NO	<0.0001
		Pm + Pa + 300 μM NO vs. Pm + 50 μM NO	0.9972
		Pm + Pa + 300 μM NO vs. Pm + Pa + 50 μM NO	0.6802
		Pm + 50 μM NO vs. Pm + Pa + 50 μM NO	0.028
	E	Pm vs. Pm + Pa	<0.0001
		Pm vs. Pm + norB::TnM	>0.9999
		Pm vs. Pm + ΔnorC	>0.9999
		Pm vs. Pm + nosZ::TnM	<0.0001
		Pm vs. Pm + ΔnarG	<0.0001
		Pm vs. Pm + ΔnirSN	<0.0001
		Pm vs. 4-bug with Pa	<0.0001
		Pm vs. 4-bug with norB::TnM	<0.0001
		Pm vs. 4-bug with ΔnorC	<0.0001
		Pm + Pa vs. Pm + norB::TnM	<0.0001
		Pm + Pa vs. Pm + ΔnorC	<0.0001
		Pm + Pa vs. Pm + nosZ::TnM	0.2816
		Pm + Pa vs. Pm + ΔnarG	<0.0001
		Pm + Pa vs. Pm + ΔnirSN	0.9952
		Pm + Pa vs. 4-bug with Pa	>0.9999
		Pm + Pa vs. 4-bug with norB::TnM	<0.0001
		Pm + Pa vs. 4-bug with ΔnorC	<0.0001
		Pm + norB::TnM vs. Pm + ΔnorC	>0.9999
		Pm + norB::TnM vs. Pm + nosZ::TnM	<0.0001
		Pm + norB::TnM vs. Pm + ΔnarG	<0.0001
		Pm + norB::TnM vs. Pm + ΔnirSN	<0.0001
		Pm + norB::TnM vs. 4-bug with Pa	<0.0001
		Pm + norB::TnM vs. 4-bug with norB::TnM	<0.0001
		Pm + norB::TnM vs. 4-bug with ΔnorC	<0.0001
		Pm + ΔnorC vs. Pm + nosZ::TnM	<0.0001
		Pm + ΔnorC vs. Pm + ΔnarG	<0.0001
		Pm + ΔnorC vs. Pm + ΔnirSN	<0.0001
		Pm + ΔnorC vs. 4-bug with Pa	<0.0001
		Pm + ΔnorC vs. 4-bug with norB::TnM	<0.0001
		Pm + ΔnorC vs. 4-bug with ΔnorC	<0.0001
		Pm + nosZ::TnM vs. Pm + ΔnarG	0.243
		Pm + nosZ::TnM vs. Pm + ΔnirSN	0.9998
		Pm + nosZ::TnM vs. 4-bug with Pa	0.9998

		Pm + nosZ::TnM vs. 4-bug with norB::TnM	<0.0001
		Pm + nosZ::TnM vs. 4-bug with ΔnorC	<0.0001
		Pm + ΔnarG vs. Pm + ΔnirSN	0.0035
		Pm + ΔnarG vs. 4-bug with Pa	0.0769
		Pm + ΔnarG vs. 4-bug with norB::TnM	<0.0001
		Pm + ΔnarG vs. 4-bug with ΔnorC	<0.0001
		Pm + ΔnirSN vs. 4-bug with Pa	>0.9999
		Pm + ΔnirSN vs. 4-bug with norB::TnM	<0.0001
		Pm + ΔnirSN vs. 4-bug with ΔnorC	<0.0001
